# Functional Characterization of Paralogous Gonadotropin-Releasing Hormone-Type and Corazonin-Type Neuropeptides in an Echinoderm

**DOI:** 10.3389/fendo.2017.00259

**Published:** 2017-09-29

**Authors:** Shi Tian, Michaela Egertová, Maurice R. Elphick

**Affiliations:** ^1^School of Biological and Chemical Sciences, Queen Mary University of London, London, United Kingdom

**Keywords:** gonadotropin-releasing hormone, corazonin, *Asterias rubens*, starfish, evolution, neuropeptide

## Abstract

Homologs of the vertebrate neuropeptide gonadotropin-releasing hormone (GnRH) have been identified in invertebrates, including the insect neuropeptide corazonin (CRZ). Recently, we reported the discovery of GnRH-type and CRZ-type signaling systems in an echinoderm, the starfish *Asterias rubens*, demonstrating that the evolutionary origin of paralogous GnRH-type and CRZ-type neuropeptides can be traced back to the common ancestor of protostomes and deuterostomes. Here, we have investigated the physiological roles of the GnRH-type (ArGnRH) and the CRZ-type (ArCRZ) neuropeptides in *A. rubens*, using mRNA *in situ* hybridization, immunohistochemistry and *in vitro* pharmacology. ArGnRH precursor (ArGnRHP)-expressing cells and ArGnRH-immunoreactive cells and/or processes are present in the radial nerve cords, circumoral nerve ring, digestive system (e.g., cardiac stomach and pyloric stomach), body wall-associated muscle (apical muscle), and appendages (tube feet, terminal tentacle). The general distribution of ArCRZ precursor (ArCRZP)-expressing cells is similar to that of ArGnRHP, but with specific local differences. For example, cells expressing ArGnRHP are present in both the ectoneural and hyponeural regions of the radial nerve cords and circumoral nerve ring, whereas cells expressing ArCRZP were only observed in the ectoneural region*. In vitro* pharmacological experiments revealed that both ArGnRH and ArCRZ cause contraction of cardiac stomach, apical muscle, and tube foot preparations. However, ArGnRH was more potent/effective than ArCRZ as a contractant of the cardiac stomach, whereas ArCRZ was more potent/effective than ArGnRH as a contractant of the apical muscle. These findings demonstrate that both ArGnRH and ArCRZ are myoexcitatory neuropeptides in starfish, but differences in their expression patterns and pharmacological activities are indicative of distinct physiological roles. This is the first study to investigate the physiological roles of both GnRH-type and CRZ-type neuropeptides in a deuterostome, providing new insights into the evolution and comparative physiology of these paralogous neuropeptide signaling systems in the Bilateria.

## Introduction

Gonadotropin-releasing hormone (GnRH) is a hypothalamic neuropeptide that regulates sexual maturation and reproduction in mammals by stimulating secretion of gonadotropins from the pituitary gland (1). Two related G-protein-coupled receptors, GnRHR and GnRHR2, mediate the physiological effects of GnRH in humans, but GnRHR2 has been lost in some mammalian lineages (e.g., mice) (2, 3). Investigation of the evolution and comparative physiology of GnRH-type signaling has revealed its occurrence and functions in non-mammalian vertebrates (3, 4). Furthermore, purification and sequencing of GnRH-type neuropeptides from the urochordate *Chelyosoma productum* provided the first definitive evidence of their occurrence in invertebrates (5). Subsequently, sequencing of the genome of the urochorate *Ciona intestinalis* enabled a detailed analysis of GnRH-type signaling in this species, with genes encoding multiple GnRH-type neuropeptide precursors and GnRH-type receptors being identified (6, 7). Functional studies provided evidence of both reproductive and non-reproductive functions of the GnRH signaling system in *C. intestinalis* (6, 8, 9).

A key breakthrough in our knowledge of the evolution of GnRH-type neuropeptide signaling was made with the discovery that a GnRH-type receptor in *Drosophila melanogaster* is activated by the insect neuropeptide adipokinetic hormone (AKH) (10). AKH is a lipid-mobilizing neuropeptide in insects that is structurally very similar to crustacean red pigment-concentrating hormone (RPCH) (11). However, AKH and RPCH share minimal sequence similarity with GnRH and thus their relationship with GnRH was not apparent when they were discovered in the 1970s (12–14). Furthermore, the existence of other AKH-like peptides in insects and other arthropods, which include corazonin (CRZ) and AKH/corazonin-related peptide (ACP), presents a more complex family of related peptides than the single GnRH peptide in humans (15, 16). Identification of receptors for CRZ and ACP has revealed that AKH receptors and ACP receptors are co-orthologs of vertebrate GnRH receptors, whereas CRZ receptors are more distantly related paralogs of AKH/ACP/GnRH-type receptors (17–19). Thus, it is proposed that a common ancestor of arthropods would have had a CRZ-type signaling pathway and an AKH/GnRH-type signaling pathway, with the latter giving rise to the AKH and ACP signaling systems by gene duplication (19).

Interestingly, analysis of genomic sequence data has revealed the occurrence of orthologs of both GnRH-type receptors and CRZ-type receptors in non-arthropodan protostomes (e.g., mollusks) and in deuterostomian invertebrates (e.g., cephalochordates and echinoderms) (18, 20–23). However, the neuropeptides that act as ligands for both receptor types were not identified in a non-arthropodan species until recently. A candidate ligand (pQILCARAFTYTHTW-NH_2_) for a CRZ-type receptor has been identified in the cephalochordate *Branchiostoma floridae* (24) but subsequent analysis has indicated that a C-terminal fragment of this peptide (FTYTHTW-NH_2_) may be the natural ligand (25).

We recently reported the identification of two neuropeptides that act as ligands for either a GnRH-type receptor or a CRZ-type receptor in an echinoderm species—the common European starfish *Asterias rubens* (25). The ligand for the *A. rubens* GnRH-type receptor has the amino acid sequence pQIHYKNPGWGPG-NH_2_ and is now known as ArGnRH. The ligand for the *A. rubens* CRZ-type receptor has the amino acid sequence HNTFTMGGQNRWKAG-NH_2_ and is now known as ArCRZ. Discovery of distinct GnRH-type and CRZ-type signaling pathways in an echinoderm has demonstrated for the first time that the evolutionarily origin of these paralogous systems can be traced to the common ancestor of protostomes and deuterostomes (26). Furthermore, there now exists a unique opportunity to investigate and compare the expression patterns and pharmacological actions of ArGnRH and ArCRZ in *A. rubens*, which may provide new insights into the evolution of GnRH/CRZ-type neuropeptide function in the animal kingdom.

The starfish *A. rubens* has been used as an experimental animal for neuropeptide research for nearly thirty years. Thus, use of antibodies to the molluscan neuropeptide FMRFamide enabled immunohistochemical visualization of the anatomy of a neuropeptidergic system in *A. rubens*, the first study of its kind in an echinoderm (27). Subsequently, neuropeptides immunoreactive with antibodies to FMRFamide-like peptides were isolated from *A. rubens* and identified as the octapeptide GFNSALMF-HN_2_ and the dodecapeptide SGPYSFNSGLTF-NH_2_ (27–29). These structurally related neuropeptides were named SALMFamide-1 (S1) and SALMFamide-2 (S2) and it has since been discovered that S1 and S2 are founding members of a family of “SALMFamides” that occur in all echinoderms (30, 31). Identification of S1 and S2 enabled functional characterization of these neuropeptides in *A. rubens* using immunohistochemistry and *in vitro* pharmacology (32–35), revealing that both peptides act as muscle relaxants. Furthermore, *in vivo* pharmacological experiments revealed that S1 and S2 induce stomach eversion in *A. rubens*, indicative of a physiological role in regulation of the extraoral feeding behavior of starfish (34).

Recently, sequencing of the neural transcriptome of *A. rubens* has enabled identification of 40 neuropeptide precursors in this species. This has provided a basis for comprehensive functional characterization of neuropeptide signaling systems in this species as a model echinoderm for neuropeptide research (36), and progress has been made with some neuropeptides. For example, the neuropeptide NGFFYamide, which is an ortholog of neuropeptide-S in vertebrates (37), triggers contraction and retraction of the stomach in *A. rubens* (38). Furthermore, use of mRNA *in situ* hybridization techniques has enabled identification of cells in *A. rubens* that produce a relaxin-like gonadotropic neuropeptide (39).

Here, we have used mRNA *in situ* hybridization and immunohistochemistry, employing novel antibodies, to investigate the expression patterns of ArGnRH and ArCRZ in *A. rubens*. This is the first study to investigate the expression of GnRH-type and CRZ-type neuropeptides at the cellular level in an echinoderm. Informed by the anatomical data, the *in vitro* pharmacological actions of ArGnRH and ArCRZ were then investigated. Collectively, the data presented here provide new insights into the evolution of GnRH/CRZ-type neuropeptide function in the animal kingdom.

## Materials and Methods

### Animals

Starfish (*A. rubens*) were collected at low tide from the Thanet coast (Kent, UK) or were obtained from a fisherman based at Whitstable (Kent, UK), and then transported to Queen Mary University of London. The starfish were maintained in an aquarium with circulating artificial seawater at ~12°C and were fed with mussels (*Mytilus edulis*). In addition, juvenile specimens of *A. rubens* (diameter 0.5–1.5 cm) were collected at the University of Gothenburg Sven Lovén Centre for Marine Infrastructure (Kristineberg, Sweden) and were fixed in Bouin’s solution for immunohistochemical analysis (see below).

### mRNA *In Situ* Hybridization

Transcripts encoding the ArGnRH precursor (ArGnRHP) and the ArCRZ precursor (ArCRZP) were first identified by analysis of *A. rubens* radial nerve cord transcriptome sequence data (36) and subsequently cDNAs comprising the complete open reading frame of ArGnRHP (accession number KT601712) and ArCRZP (accession number KT601713) have been cloned and sequenced (25). These cDNAs were used here for production of digoxigenin-labeled antisense and sense probes for ArGnRHP and ArCRZP, employing methods reported previously (39, 40). The templates for generation of the ArGnRHP and ArCRZP probes were 490 and 316 bases in length, respectively, and the probes were used without being hydrolyzed. Specimens of *A. rubens* were fixed, decalcified, and sectioned and then slide-mounted sections were processed for mRNA *in situ* hybridization using methods reported previously (39). To facilitate interpretation of staining, sections of *A. rubens* stained using Masson’s Trichrome method were also prepared, employing methods reported previously (41). Images of stained sections were captured with a QIClick™ CCD Color Camera (Qimaging) linked to a DMRA1 light microscope (Leica), using Volocity^®^ v.6.3.1 image analysis software (PerkinElmer) installed on an iMac (27-in., Late 2013 model with OXS Yosemite, Version 10.10). Adobe Photoshop CC (version 14.0, ×64) was used for removing dust, contrast adjustment, cropping images, and assembling images into montages.

### Production and Characterization of Antibodies to ArGnRH and ArCRZ

To generate rabbit antisera to ArGnRH (pQIHYKNPGWGPG-NH_2_) a C-terminal fragment of ArGnRH (KNPGWGPG-NH_2_) was used as a peptide antigen. To generate rabbit antisera to ArCRZ (HNTFTMGGQNRWKAG-NH_2_) two different peptides were tested as antigens. First, a peptide comprising the C-terminal region of ArCRZ (KYGQNRWKAG-NH_2_), with an N-terminal lysine residue incorporated to provide two reactive amine groups for coupling to a carrier protein (thyroglobulin). A tyrosine residue was also incorporated at the N-terminus so that the peptide could, if needed, be radiolabeled with iodine-125 (e.g., for use in a radioimmunoassay). Second, an analog of the C-terminal region of ArCRZ (KYGQNRWRAG-NH_2_) in which the lysine corresponding to the thirteenth residue of ArCRZ was replaced with arginine. The rationale for testing this second antigen peptide was a concern that chemical reaction of the free amine group in the lysine side chain with glutaraldehyde used for production of antigen peptide–carrier protein conjugates (see below) may affect antigenicity. The antigen peptides were synthesized by Peptide Protein Research Ltd. (Hampshire, UK) and were conjugated to the carrier protein thyroglobulin (Sigma-Aldrich) using 5% glutaraldehyde (v/v) in phosphate buffer (PB, 0.1 M sodium phosphate dibasic and 0.1 M sodium phosphate monobasic, pH = 7.2) as a coupling reagent. Peptide–thyroglobulin conjugates were then dialyzed using phosphate-buffered saline (PBS), aliquoted, and stored at −20°C.

Rabbit immunization and serum collection was performed by Charles River Labs (Margate, England, UK) according to the following protocol. On day 0, preimmune serum was collected and the first immunization (~100 nmol of conjugated antigen peptide in Freund’s complete adjuvant) was administered. Booster immunizations (~50 nmol of conjugated antigen peptide in Freund’s incomplete adjuvant) were administered on days 28, 42, and 56. Antiserum samples were collected on days 37 and 51 and a final bleed was collected on day 70.

To assess production of antibodies during the immunization protocol and following collection of a terminal bleed, antisera were tested for antibodies to the antigen peptides using enzyme-linked immunosorbent assay (ELISA). 100 µl of a 1 µM solution of antigen peptide dissolved in carbonate/bicarbonate buffer (25 mM anhydrous sodium carbonate, 25 mM sodium bicarbonate, pH 9.8) was pipetted into wells of a PVC microtiter plate (Starlab), which was then covered with Parafilm M^®^ (Starlab) and incubated overnight at 4°C. The following day the liquid contents of the plate were disposed of, the wells were rinsed with 200 µl PBS (3×, 10 min), and then the plate was drained on blotting paper. Then 200 µl of a blocking solution containing 5% goat antiserum/PBS was added to each well and left at room temperature for 2 h. The blocking buffer was discarded and each well was washed with PBS containing 0.1% Tween-20 (PBST; 200 µl; 3×, 10 min) and then the plate was drained, as above. Varying dilutions of preimmune serum or antiserum (10^−3^ to 10^−8^) diluted in 5% goat serum (Sigma-Aldrich)/PBS was added to each well and incubated overnight at 4°C. The antiserum was discarded and each well was washed with PBST (200 µl; 3×, 10 min) and then the plate was drained. Alkaline phosphatase-conjugated goat anti-rabbit IgG secondary antibodies (ThermoFisher Scientific; diluted 1:3,000 in 5% goat antiserum/PBST) were added to each well and incubated for 3 h at room temperature. After washing the plate with PBST (4×, 10 min), 100 µl p-nitrophenylphosphate alkaline phosphatase substrate (Vector Laboratories) made up in carbonate/bicarbonate buffer was added to each well and then after a 20 min incubation at room temperature absorbance at 415 nm was measured using FLUOstar Omega (BMG LABTECH). Mean absorbance values were calculated and plotted using Prism 6.0c.

Antibodies to the antigen peptide were affinity-purified using AminoLink^®^ Plus Immobilization Kit (Thermo Scientific). 1 mg of the ArGnRH/ArCRZ antigen peptides were dissolved in 2 ml of PBS and coupled to a column of beaded agarose, according to the manufacturer’s instruction. After washing with 5 ml of PBS (2×), 1 ml of ArGnRH antiserum or ArCRZ antiserum was added to the column and incubated at room temperature for 2 h on a rocking table. After washing with 5 ml of PBS (6×), bound antibodies were eluted using 7 ml of glycine elution buffer [6.3 ml of 100 mM glycine (VWR) and 0.7 ml of Tris (1 M, pH = 7.0)] and then 7 ml of triethylamine elution buffer [6.3 ml of 100 mM triethylamine (Sigma-Aldrich) and 0.7 ml of Tris (1 M, pH = 7.0)]. The glycine eluate and triethylamine eluate were then dialyzed separately in PBS at 4°C for at least 48 h. After addition of 0.1% sodium azide (VWR), the dialyzed antibodies were stored at 4°C.

### Immunohistochemistry

Specimens of *A. rubens* (diameter 0.5–6 cm) were fixed in Bouin’s solution (75% saturated picric acid in seawater, 25% formalin, 5% acetic acid) for at least 2 days, and decalcified with 2% ascorbic acid/0.15 M sodium chloride solution at 4°C for at least 1 week. The arms and central disk regions of decalcified larger specimens of starfish (>2 cm diameter) were separated and processed separately, whereas smaller specimens were processed intact. Starfish tissue samples were embedded in paraffin, sectioned at 8 µm (RM 2145 microtome, Leica) and mounted on chrome alum/gelatin coated microscope slides. Paraffin was removed by immersion of slides in xylene (3× 10 min) and then slides were immersed in 100% ethanol (3× 10 min). Endogenous peroxidase activity was quenched by placing slides in 0.3% hydrogen peroxide (VWR Chemicals) in methanol for 30 min. The tissue sections were then rehydrated through a series of 10 min ethanol washes (90, 70, and 50%) and finally rinsed in distilled water. Slides were blocked in 5% goat serum (Sigma-Aldrich) made up in PBST (2 h at room temperature) and then were incubated overnight at 4°C in affinity-purified primary antibodies diluted 1:5 or 1:10 in 5% goat serum/PBST. Following a series of washes in PBST, peroxidase-conjugated AffiniPure Goat anti-rabbit IgG (Jackson ImmunoResearch Laboratories, West Grove, PA, USA) were used as secondary antibodies (1:3,000 dilution, in 2% goat serum/PBST) and were added to each slide (500 μl/slide) and incubated for 2 h at room temperature. Immunostaining was visualized using staining buffer [0.05% diaminobenzidine (VWR Chemicals), 0.05% nickel chloride, 0.015% hydrogen peroxide in PBS]. Slides were then washed in autoclaved distilled water when staining was observed. Following dehydration through washes in autoclaved distilled water, 50% ethanol (1× 10 min), 70% ethanol (1× 10 min), 90% ethanol (1× 10 min), and 100% ethanol (2× 10 min), slides were washed in xylene (2× 10 min) before being mounted with coverslips using DPX (Thermo Scientific) as a mounting medium. Control experiments were performed using antibodies preabsorbed with ArGnRH antigen peptide (Peptide Protein Research Ltd., Hampshire, UK). Preabsorption was done by incubating affinity-purified antibodies with 200 µM antigen peptide for 1–2 h at room temperature. Then the preabsorbed antibodies were diluted to 1:5 or 1:10 in PBS and tested as above. Images of immunostained sections were captured with a QIClick™ CCD Color Camera (Qimaging) linked to a DMRA2 light microscope (Leica), using Volocity^®^ v.6.3.1 image analysis software (PerkinElmer) installed on an iMac (27-in., Late 2013 model with OS X Yosemite, Version 10.10). Adobe Photoshop CC (version 14.0, ×64) was used for removing dust, contrast adjustment, cropping images, and assembling images into montages.

### *In Vitro* Pharmacology

The predicted structures of ArGnRH (pQIHYKNPGWGPG-NH_2_) and ArCRZ (HNTFTMGGQNRWKAG-NH_2_) have been confirmed by mass spectrometric analysis of *A. rubens* radial nerve cord extracts (25). Accordingly, ArGnRH and ArCRZ were custom synthesized (Peptide Protein Research Ltd., Hampshire, UK) to enable testing of the pharmacological effects of these neuropeptides. Both peptides were tested on *in vitro* preparations of cardiac stomach, apical muscle, and tube foot dissected from at least five different specimens of *A. rubens*. The cardiac stomach was dissected as described previously (32, 38) and then cotton ligatures were tied around the esophagus and the aboral side of the cardiac stomach. The apical muscle was dissected from the aboral body wall of the arms of *A. rubens*, cut into ~1 cm long segments and tied at each end with cotton ligatures, as described previously (35). The tube foot preparations were dissected from starfish arms by cutting out a square-shaped piece of ambulacral body wall and removing all of the tube feet except one at the center, with its ampulla intact. The external epithelium of the tube foot was scraped off using a blunt scalpel blade to facilitate penetration of peptides when tested *in vitro* (see below) and then cotton ligatures were tied around the ambulacral body wall and the tube foot sucker, as described previously (35). The dissected preparations were tied at one end to a fixed metal hook in a 20 ml aerated organ bath containing circulating artificial sea water at ~11°C. The other ligature was attached to a high grade Isotonic Transducer (ADinstruments MLT0015), which was connected *via* a bridge amplifier (FE221 Bridge Amp, ADInstruments Pty Ltd.) to data acquisition hardware (Power Lab 2/36, AD Instruments Pty Ltd.). Data were collected and analyzed using LabChart (v8.0.7) software installed on a laptop computer (Lenovo E540, Windows 7 Professional).

Once set up in the organ bath, preparations were incubated in artificial seawater, with several washes until a stable baseline length was achieved. Then synthetic peptides (ArGnRH or ArCRZ) were added to the organ bath to achieve concentrations ranging from 10^−6^ to 10^−10^ M. Known contractants of the preparations tested, NGFFYamide (for cardiac stomach, 10^−7^ M) (38) or acetylcholine (ACh; for apical muscle and tube foot preparations, 10^−6^ M), were tested to check the viability of preparations and to enable normalization of responses to ArGnRH or ArCRZ in different experiments. Thus, the effects of ArGnRH or ArCRZ on cardiac stomach preparations were normalized to the maximal effect observed with NGFFYamide (10^−7^ M) and the effects of ArGnRH or ArCRZ on apical muscle and tube foot preparations were normalized to the maximal effect observed with ACh (10^−6^ M). Dose–response curves were generated using the non-linear regression (curve fit) model in Prism 6 (GraphPad 6.0c). The maximal response (*E*_max_) was expressed as the percentage (%) of the maximal contraction induced by each peptide compared to the maximal contraction induced by 10^−7^ M of NGFFYamide (for cardiac stomach) or 10^−6^ M of ACh (for apical muscle and tube foot preparations). Statistical analysis was performed using *t*-tests when carrying out pairwise comparisons between peptides tested at the same concentration.

### *In Vivo* Pharmacology

Twenty specimens of *A. rubens*, which had been withheld from a food supply for 1 week, were used to investigate if ArGnRH and ArCRZ can induce cardiac stomach retraction *in vivo*. As reported previously (38), cardiac stomach eversion was induced by placing starfish in a glass tank containing 2% magnesium chloride (MgCl_2_) dissolved in seawater, which acts as a muscle relaxant in marine invertebrates (42). Then 10 µl of 1 mM ArGnRH or ArCRZ peptide was injected into the perivisceral celom at two sites in the aboral body wall of the arms proximal to their junctions with the central disk, as reported previously (38). Starfish injected with ArGnRH or ArCRZ were video recorded for 5 min. Then as a positive control, NGFFYamide (10 µl of 100 nM), which is a potent cardiac stomach contractant *in vitro* and *in vivo* (38), was injected into the perivisceral celom (at two sites in the aboral body wall different to those used for ArGnRH or ArCRZ) and the starfish were video recorded for a further 5 min.

## Results

### Anatomy of the Starfish *A. rubens*

**Figure 1:**
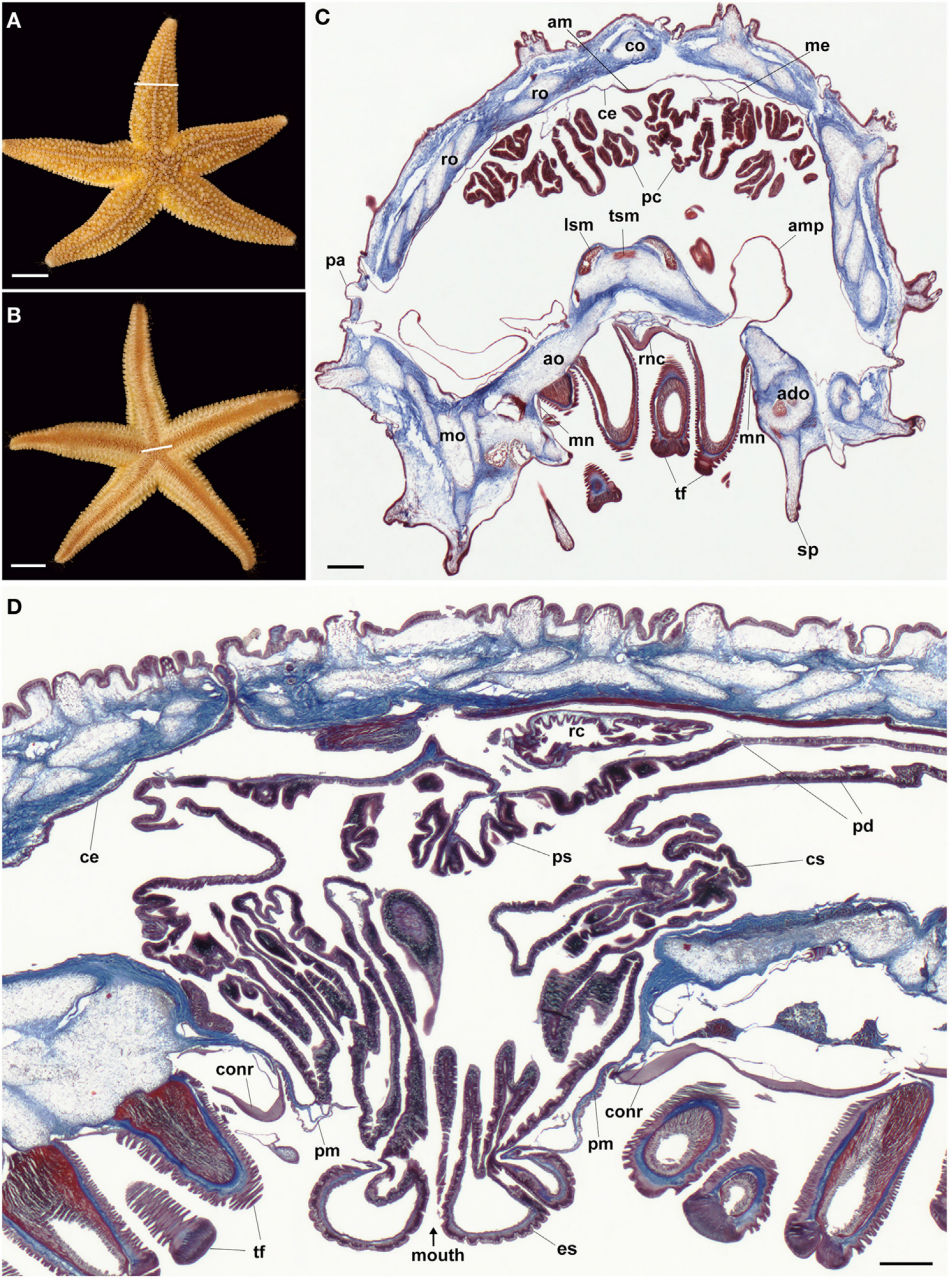
Anatomy of the starfish *Asterias rubens*. **(A)** Aboral view of a specimen of *A. rubens*; the white line indicates the approximate position of the section shown in **(C)**. **(B)** Oral view of a specimen of *A. rubens*; the white line indicates the approximate position of the section shown in **(D)**. **(C)** Trichrome-stained transverse section of an arm from a specimen of *A. rubens*. The body wall comprises ossicles (which appear white here) that are surrounded and interlinked by collagenous tissue (stained blue). The body wall has several types of appendages, which include spines and papulae. The body wall is lined internally by a coelomic epithelium, which is underlain along its midline aborally by a longitudinally orientated muscle known as the apical muscle. Linked to the aboral body wall by mesenteries are digestive organs known as pyloric caeca. The oral side of the body wall comprises ambulacral and adambulacral ossicles and two rows of tube feet, which are linked to bulb-shaped ampullae located internal to the body wall. The V-shaped radial nerve cord runs between the two rows of paired tube feet; lateral to the outer rows of tube feet are the marginal nerves. **(D)** Trichrome-stained transverse section of the central disk region, which is largely filled by the highly folded cardiac stomach. The cardiac stomach is linked orally to a short esophagus, which in this preparation is everted through the oral opening (mouth) that is surrounded by a peristomial membrane. Lateral to the peristomial membrane is the cirumoral nerve ring. Aboral to the cardiac stomach is the smaller pyloric stomach, which is linked *via* pyloric ducts to the pyloric caeca located in the arms [see **(C)**]. Aboral to the pyloric stomach is a short rectum (not seen here), which has associated rectal caeca. Abbreviations: ado, adambulacral ossicle; am, apical muscle; ao, ambulacral ossicle; ce, coelomic epithelium; co, carinal ossicle; conr, circumoral nerve ring; cs, cardiac stomach; es, esophagus; lsm, longitudinal supra-ambulacral muscle; me, mesentery; mn, marginal nerve; mo, marginal ossicle; pa, papula; pc, pyloric caeca; pd, pyloric duct; pm, peristomial membrane; ps, pyloric stomach; rc, rectal caeca; ro, reticular ossicle; sp, spine; tf, tube foot; tsm, transverse supra-ambulacral muscles. Scale bars: **(A,B)** = 1 cm; **(C,D)** = 210 µm.

**Figure 2:**
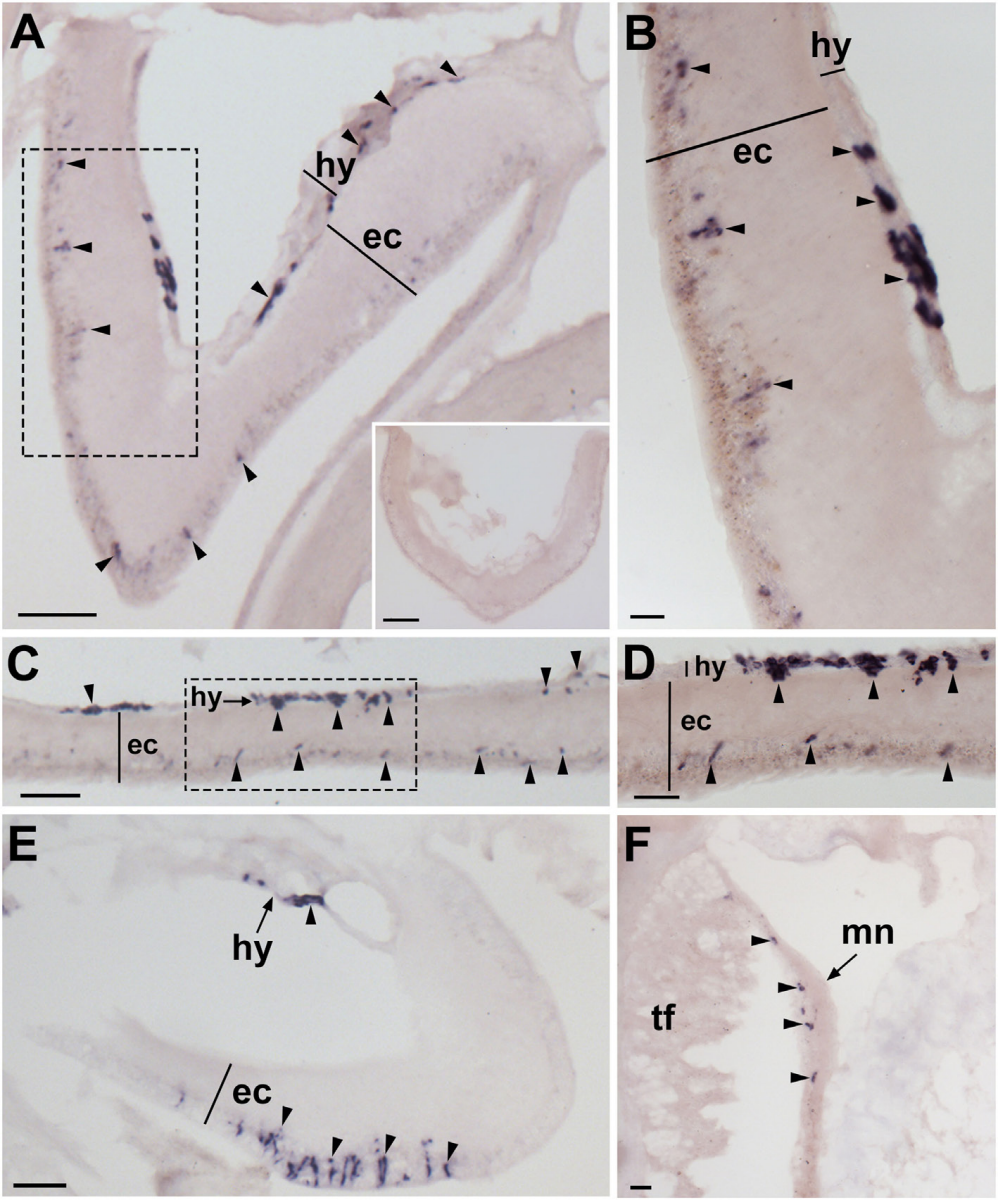
Localization of *Asterias rubens* gonadotropin-releasing hormone precursor (ArGnRHP) mRNA in the nervous system of *A. rubens* using *in situ* hybridization. **(A,B)** Transverse section of a radial nerve cord incubated with antisense probes, showing stained cells (arrowheads) in both the hyponeural and ectoneural regions. The inset of **(A)** shows absence of staining in a transverse section of a radial nerve cord incubated with sense probes, demonstrating the specificity of staining observed with antisense probes. A higher magnification image of the boxed area is shown in **(B)**. **(C)** Longitudinal parasagittal section of a radial nerve cord showing stained cells (arrowheads) in both the hyponeural and ectoneural regions. A higher magnification image of the boxed area is shown in **(D)**. **(E)** Transverse section of the central disk region showing stained cells (arrowheads) in both the hyponeural and the ectoneural regions of the circumoral nerve ring; note, however, that during tissue processing the hyponeural region has been displaced from its natural position adjacent to the ectoneural region. **(F)** Stained cells (arrowheads) in the marginal nerve, which is located lateral to the outer row of tube feet on each side of the arms. Abbreviations: ec, ectoneural region; hy, hyponeural region; mn, marginal nerve; tf, tube foot. Scale bars: [**(A)**, inset, **(C,E)**] = 50 µm; **(B)** = 10 µm; **(D)** = 20 µm; **(F)** = 30 µm.

**Figure 3:**
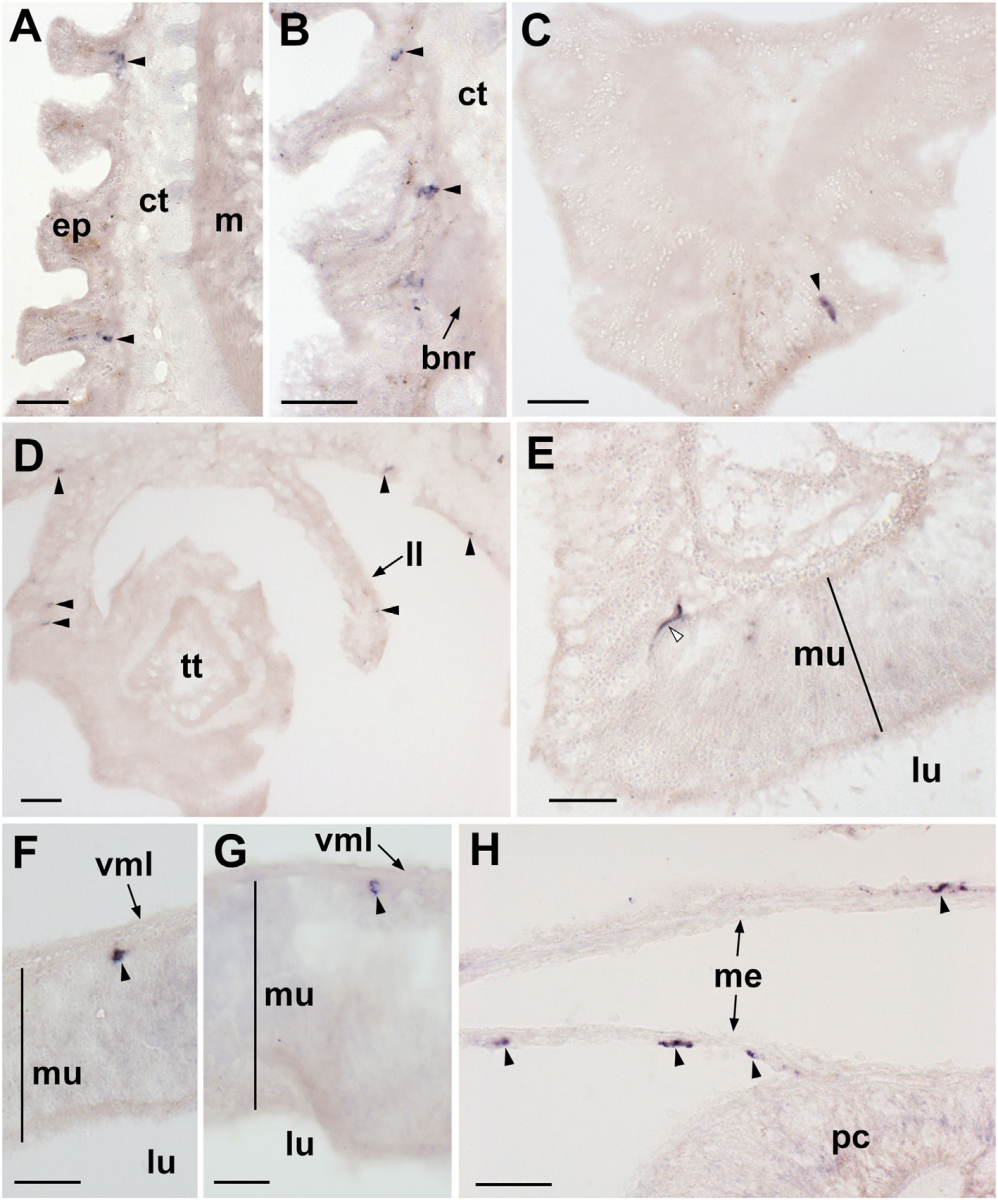
Localization of *Asterias rubens* gonadotropin-releasing hormone precursor (ArGnRHP) mRNA in the tube feet, terminal tentacle and digestive system of *A. rubens* using *in situ* hybridization. **(A)** Longitudinal section of a tube foot showing stained cells (arrowheads) in the subepithelial layer of the tube foot stem. **(B)** Stained cells (arrowheads) located in the subepithelial layer above the tube foot sucker and near the basal nerve ring. **(C)** Stained cell (arrowhead) located in the optic cushion, which is located at the base of the terminal tentacle. **(D)** Stained cells (arrowheads) located in a lateral lappet and the body wall epithelium surrounding the terminal tentacle. **(E)** Stained cell (white arrowhead) in the mucosal layer of the cardiac stomach. **(F)** Stained cell (arrowhead) located close to the basiepithelial nerve plexus of the cardiac stomach. **(G)** Stained cell (arrowhead) located close to the basiepithelial nerve plexus of the pyloric stomach. **(H)** Stained cells (arrowheads) located in the mesenteries associated with the pyloric caeca. Abbreviations: bnr, basal nerve ring; ct, collagenous tissue; ep, epidermis; ll, lateral lappet; lu, lumen; m, muscle; me, mesentery; mu, mucosa; pc, pyloric cecum; vml, visceral muscle layer. scale bars: **(A,B,D,H)** = 30 µm; **(C,E,F,G)** = 20 µm.

**Figure 4:**
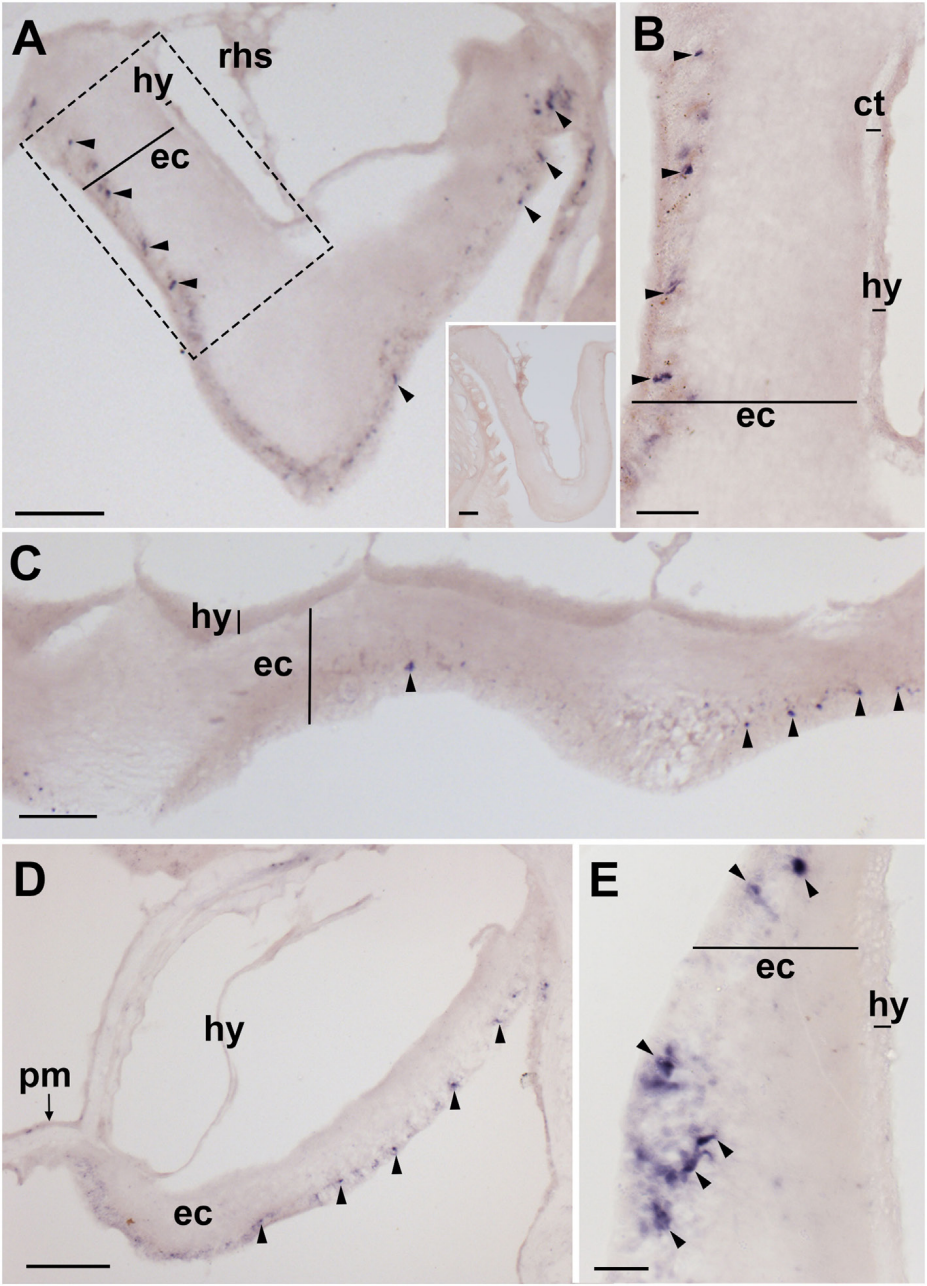
Localization of *Asterias rubens* corazonin precursor (ArCRZP) mRNA in the nervous system of *A. rubens* using *in situ* hybridization. **(A,B)** Transverse section of a radial nerve cord incubated with antisense probes, showing stained cells (arrowheads) in the ectoneural region but not in the hyponeural region. The inset of **(A)** shows absence of staining in a transverse section of a radial nerve cord incubated with sense probes, demonstrating the specificity of staining observed with antisense probes. A higher magnification image of the boxed area is shown in **(B)**. **(C)** Longitudinal parasagittal section of a radial nerve cord showing stained cells (arrowheads) in the ectoneural region but not in the hyponeural region. **(D)** Transverse section of the central disk region showing stained cells (arrowheads) in the ectoneural region of the circumoral nerve ring but not in the hyponeural region, which has been displaced from its natural position adjacent to the ectoneural region during tissue processing. **(E)** High magnification image of the stained cells (arrowheads) in the ectoneural region of the circumoral nerve ring. Abbreviations: ct, collagenous tissue; ec, ectoneural region; hy, hyponeural region; pm, peristomial membrane; rhs, radial hemal strand. Scale bars: [**(A)** inset **(C)**] = 60 µm; **(B,E)** = 20 µM; **(D)** = 120 µm.

**Figure 5:**
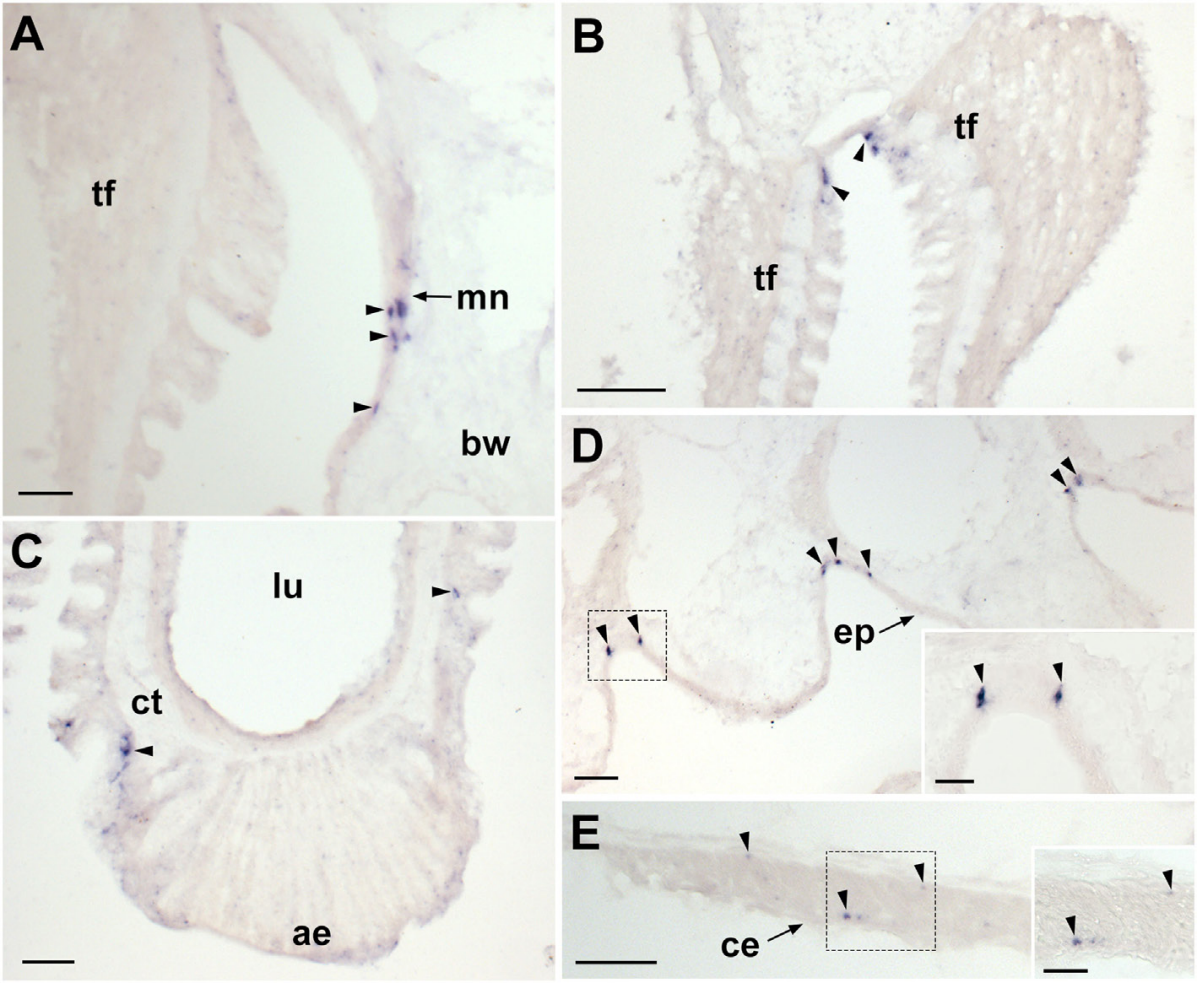
Localization of *Asterias rubens* corazonin precursor (ArCRZP) mRNA in the marginal nerve, tube foot and body wall-associated structures of *A. rubens* using *in situ* hybridization. **(A)** Stained cells in the marginal nerve. **(B)** Stained cells (arrowheads) located within or beneath the external epithelium layer at the junction between adjacent tube feet. **(C)** Stained cells (arrowheads) located near to the tube foot sucker. **(D)** Stained cells (arrowheads) located in the external epidermis on the oral side of the body wall. **(E)** Transverse section of the apical muscle showing stained cells (arrowheads) in the coelomic epithelium and close to the circular muscle layer. The boxed region of **(E)** is shown at higher magnification in the inset. Abbreviations: ae, adhesive epidermis of tube foot sucker; bw, body wall; ct, collagenous tissue; lu, lumen; mn, marginal nerve; tf, tube foot. Scale bars: **(A,E,D)** = 50 µm; **(B)** = 100 µm; **(C)** = 60 µm; [**(D,E)** insets] = 20 µm.

**Figure 6:**
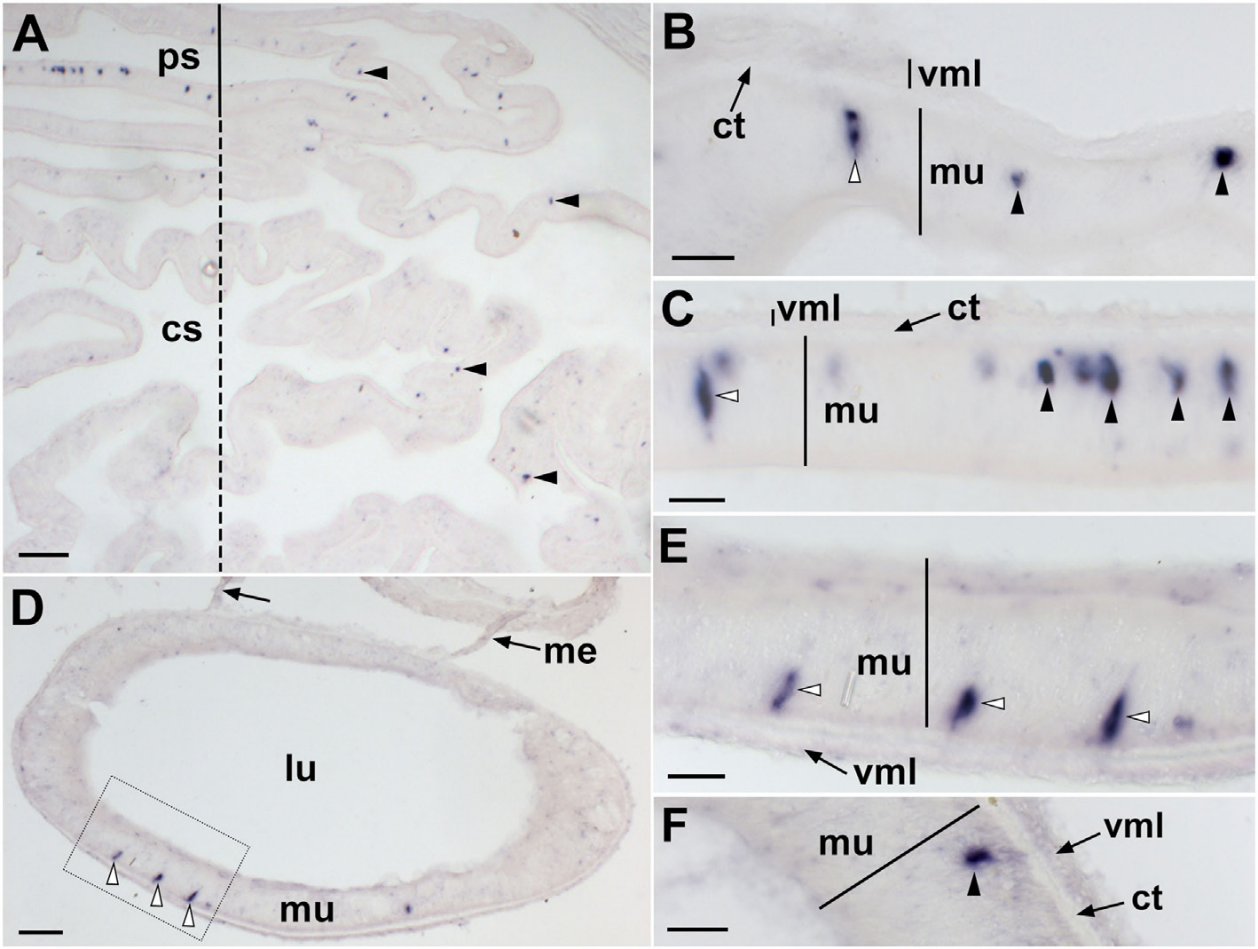
Localization of *Asterias rubens* corazonin precursor (ArCRZP) mRNA in the digestive system of *A. rubens* using *in situ* hybridization. **(A)** Transverse section through the central disk region showing stained cells (arrowheads) in the cardiac stomach (dashed line) and pyloric stomach (solid line). Stained cells are more abundant in the pyloric stomach and in the aboral (uppermost) region of the cardiac stomach than in the oral (lowermost) region of the cardiac stomach. **(B)** High magnification image showing stained cells in the cardiac stomach located both in the mucosal layer (white arrowhead) and close to the basiepithelial nerve plexus (black arrowheads). **(C)** High magnification image showing stained cells in the pyloric stomach located both in the mucosal layer (white arrowhead) and close to the basiepithelial nerve plexus (black arrowheads). **(D)** Transverse section of a pyloric duct showing stained cells in the oral (lowermost) region. The boxed area is shown at higher magnification in **(E)**. **(E)** High magnification image of a pyloric duct showing stained cells in the mucosal layer (white arrowheads). **(F)** Stained cells (arrowhead) located close to the basiepithelial nerve plexus of a pyloric duct. Abbreviations: cs, cardiac stomach; ct, collagenous tissue; lu, lumen; me, mesentery; mu, mucosa; ps, pyloric stomach; vml, visceral muscle layer. Scale bars: **(A)** = 100 µm; **(B,C,E,F)** = 20 µm; **(D)** = 60 µm.

**Figure 7:**
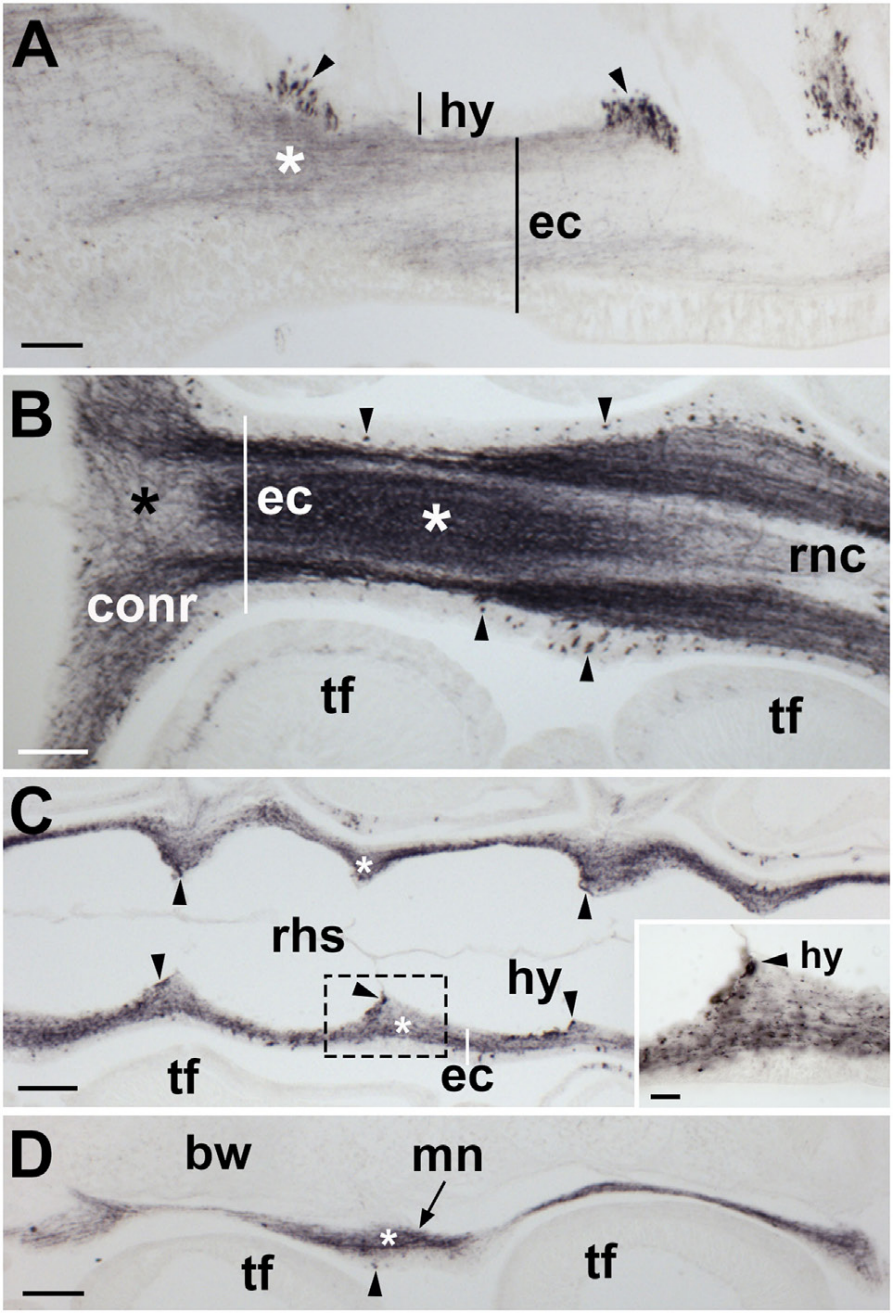
Localization of *Asterias rubens* gonadotropin-releasing hormone (ArGnRH) immunoreactivity in the nervous system of *A. rubens*. **(A)** Longitudinal section of a radial nerve cord showing immunostaining in both the hyponeural and ectoneural regions. Clusters of stained cells (arrowheads) can be seen in the hyponeural region. Immunostained fibers (asterisk) can be seen in neuropile of the ectoneural region. **(B)** Horizontal section through the radial nerve cord and circumoral nerve ring of a juvenile specimen. Immunostained cell bodies (arrow heads) can be seen in the epithelial layer of the ectoneural region. The neuropile of the radial nerve cord and circumoral nerve ring contains a meshwork of immunostained fibers but the density of the stained fibers varies, with a higher density in some areas (white asterisk) and a lower density in other areas (black asterisk). **(C)** Horizontal section of a radial nerve cord [aboral to the section shown in **(B)**] showing the segmental structure of the radial nerve cord. Immunostained fibers can be seen in the ectoneural region (asterisk) and immunostained cells (arrowheads) can be seen in the hyponeural region. The boxed region is shown at higher magnification in the inset. **(D)** Longitudinal section of the marginal nerve showing immunostained cells (arrowhead) and processes (asterisk). Abbreviations: bw, body wall; conr, circumoral nerve ring; ec, ectoneural region; hy, hyponeural region; mn, marginal nerve; rnc, radial nerve cord; rhs, radial hemal strand; tf, tube foot. Scale bars: **(A–D)** = 60 µm; [**(C)** inset] = 20 µm.

**Figure 8:**
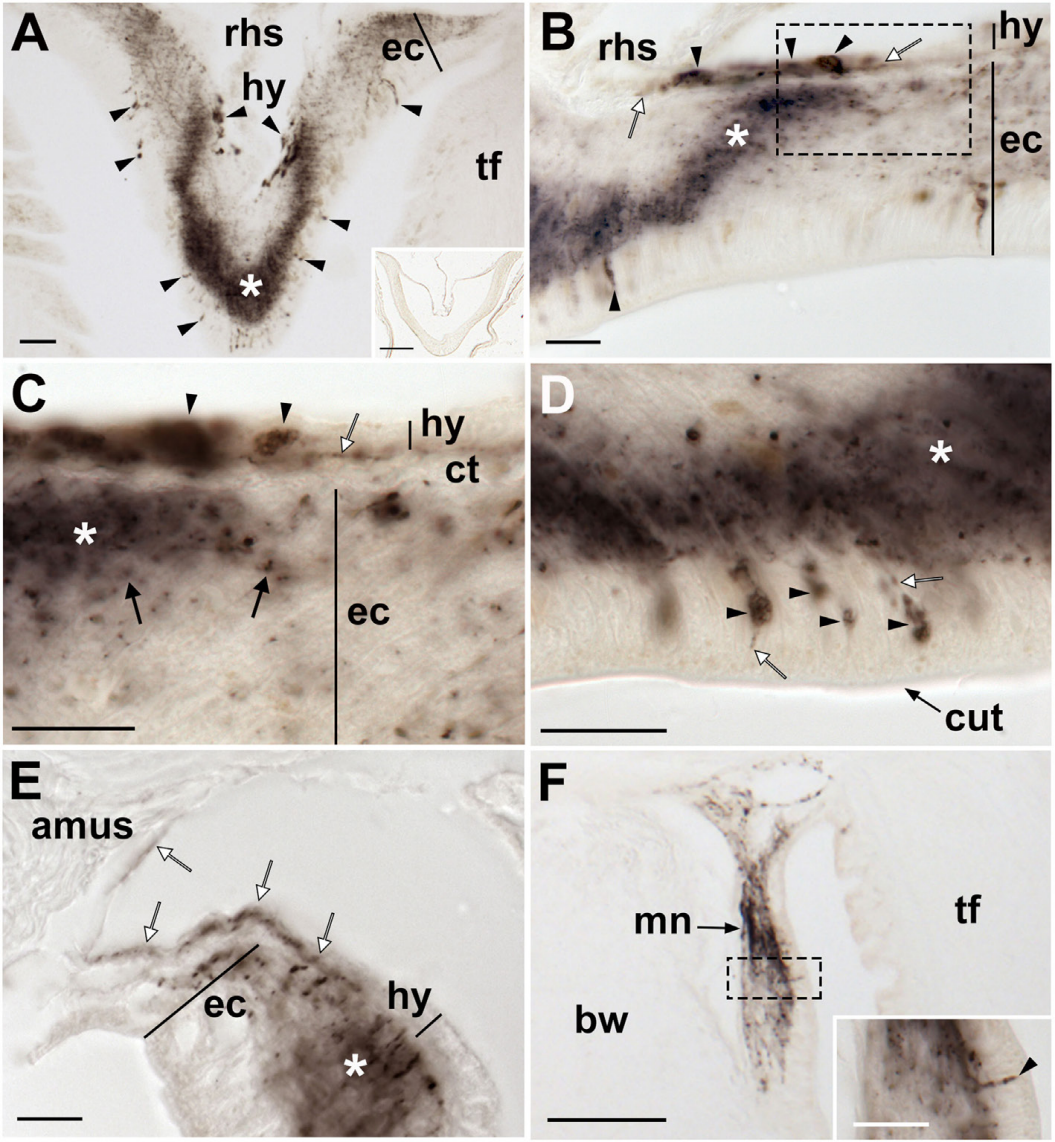
Localization of *Asterias rubens* gonadotropin-releasing hormone (ArGnRH) immunoreactivity in the radial nerve cords and marginal nerves of *A. rubens*. **(A)** Transverse section of a radial nerve cord showing stained cells (arrowheads) in both the hyponeural and ectoneural regions. The intensity of immunostaining in the neuropile of the ectoneural region varies, with the region at the apex of the V-shaped nerve cord intensely stained (asterisk) and the lateral regions less intensely stained. The inset shows absence of immunostaining in a radial nerve cord section incubated with affinity-purified ArGnRH-antibodies preabsorbed with the antigen peptide (ArGnRH-ag), demonstrating the specificity of immunostaining observed with the ArGnRH antibody. **(B)** High magnification image of a radial nerve cord showing immunostained cell bodies in the hyponeural and ectoneural regions. Note that in the neuropile of the ectoneural region there is a band of immunostaining (white asterisk), which is located opposite immunostained hyponeural cell bodies. The boxed area is shown at higher magnification in **(C)**. **(C)** Immunostained processes (white arrow) derived from monopolar cell bodies (arrowheads) can be seen here in the hyponeural region. Stained axonal profiles (black arrows) can be seen at the margin of the intensely stained region of the ectoneural neuropile (asterisk). The layer of collagenous tissue that separates the hyponeural and ectoneural regions is unstained. **(D)** High magnification image of the ectoneural region of a radial nerve cord showing immunostained bipolar-shaped cells in the subcuticular epithelium (arrowhead). Immunostained processes extending into the ectoneural neuropile and toward the cuticular layer (white arrows) can also be seen here. **(E)** High magnification image showing immunostaining in the lateral region of a radial nerve cord, with staining in the ectoneural neuropile (asterisk) and in the processes of hyponeural neurons that project from the radial nerve cord around the lateral wall of the radial perihemal canal (white arrows). **(F)** Immunostaining in the marginal nerve. The boxed area is shown at higher magnification in the inset, where a stained bipolar-shaped cell can be seen in the epithelial layer (arrowhead) and stained processes can be seen in the underlying neuropile. Abbreviations: amus, ambulacral muscle; ct, collagenous tissue; cut, cuticular layer; ec, ectoneural region; hy, hyponeural region; rhs, radial hemal strand; tf, tube foot. Scale bars: **(A,F)** = 50 µm; [**(A)** inset] = 120 µm; [**(B–F)** inset] = 20 µm.

**Figure 9:**
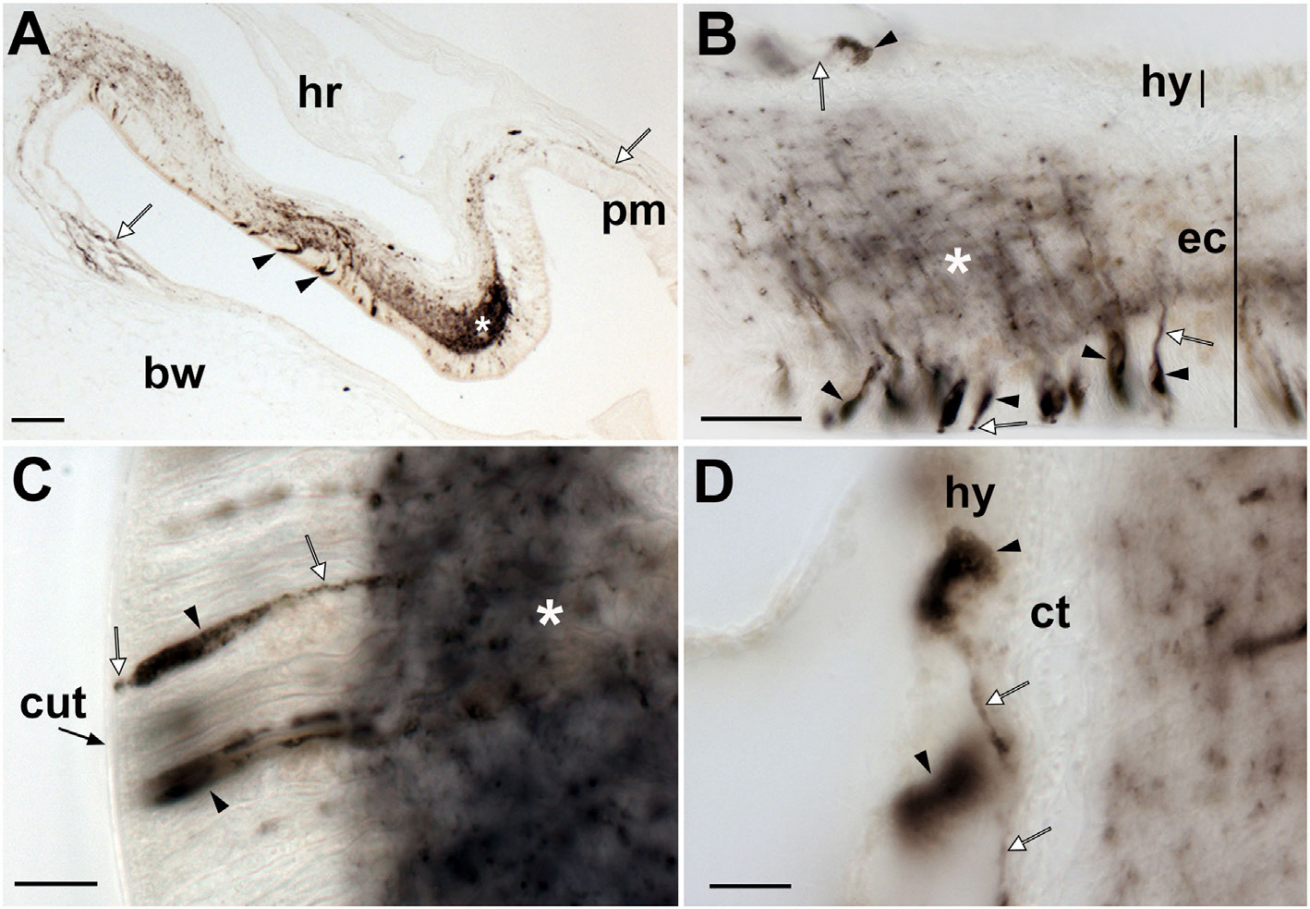
Localization of *Asterias rubens* gonadotropin-releasing hormone (ArGnRH) immunoreactivity in the circumoral nerve ring of *A. rubens*. **(A)** Immunostaining in a transverse section of a circumoral nerve ring, with immunostained processes projecting from the circumoral nerve ring into the peristomial membrane and the adjacent body wall (white arrows). A cluster of immunostained cells can be seen in the ectoneural epithelium of the medial region of the nerve ring (arrowheads) and an intensely stained region of ectoneural neuropile (asterisk) can be seen at the apex of the nerve ring. **(B)** High magnification image of a circumoral nerve ring showing immunostained cells in the hyponeural and ectoneural regions (arrowheads) with stained processes (white arrows). Immunostained processes of ectoneural cells project into the underlying neuropile (white asterisk). **(C)** High magnification image of the ectoneural region showing immunostained bipolar-shaped cells in the subcuticular epithelium (arrowheads) with immunostained processes (white arrows) extending toward the cuticular layer and the intensely stained neuropile (white asterisk). **(D)** High magnification image of the hyponeural region showing immunostained monopolar-shaped cells (arrowhead) with immunostained processes (white arrows) adjacent to the unstained collagenous tissue layer. Abbreviations: bw, body wall; ct, collagenous tissue; cut, cuticular layer; ec, ectoneural region; hr, hemal ring; hy, hyponeural region; pm, peristomial membrane. Scale bars: **(A)** = 50 µm; **(B)** = 20 µm; **(C,D)** = 10 µm.

**Figure 10:**
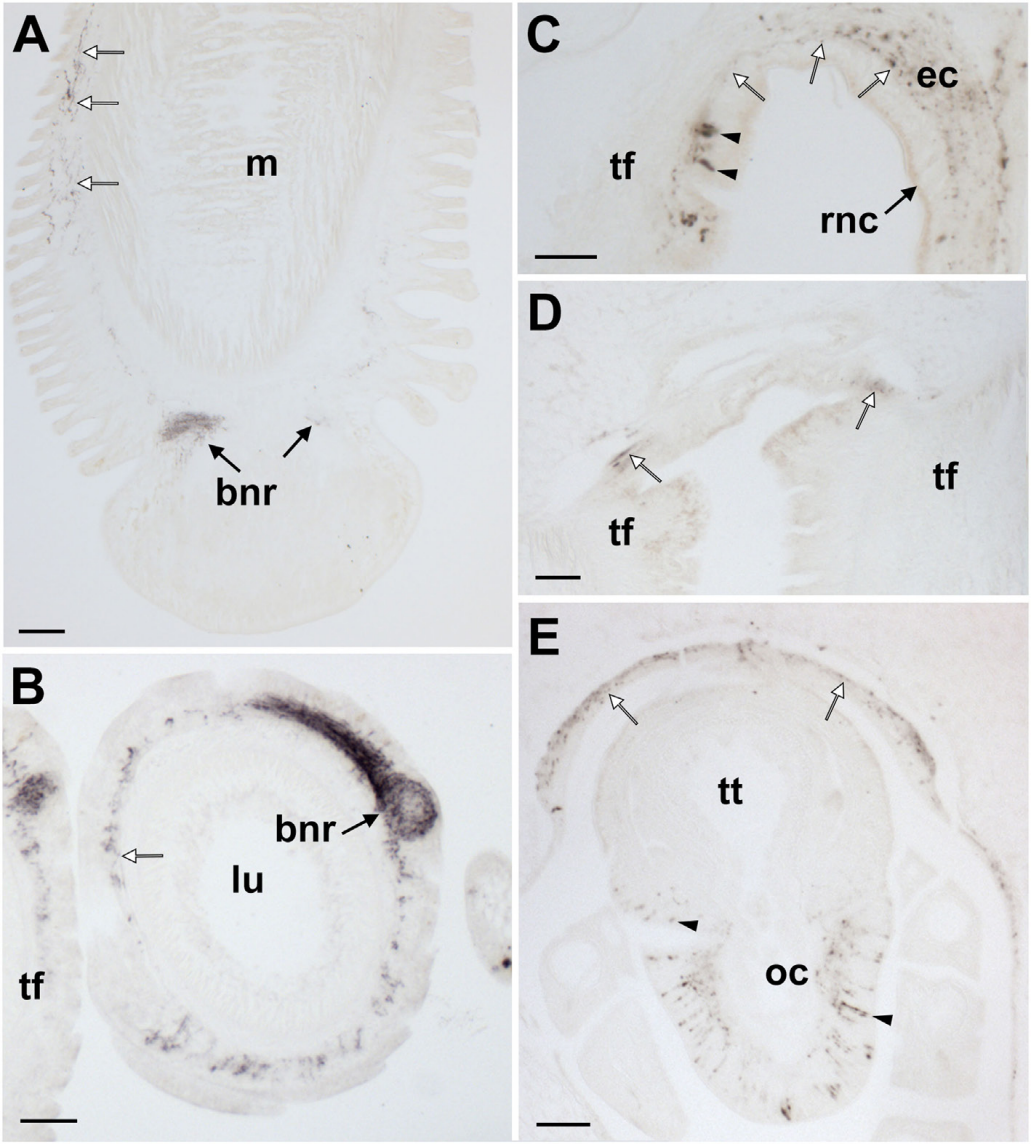
Localization of *Asterias rubens* gonadotropin-releasing hormone (ArGnRH) immunoreactivity in the tube feet and terminal tentacle of *A. rubens*. **(A)** Longitudinal section of a tube foot showing immunostaining in the subepithelial nerve plexus (white arrows) and basal nerve ring (black arrows). **(B)** Transverse section of a tube foot showing immunostained processes in the subepithelial nerve plexus (white arrow) and in the basal nerve ring (black arrow). **(C)** Immunostained cells (arrowheads) and processes (white arrows) at the junction between a tube foot and the radial nerve cord. **(D)** Immunostaining at the junction between adjacent tube feet. **(E)** Immunostained cells (arrowheads) and processes in the optic cushion, which is located at the base of the terminal tentacle. Immunostained processes (white arrows) can also be seen here in the basiepithelial plexus of the body wall epithelium surrounding the terminal tentacle. Abbreviations: bnr, basal nerve ring; ct, collagenous tissue; ec, ectoneural region; lu, lumem; m, muscle; oc, optic cushion; rnc, radial nerve cord; tf, tube foot; tt, terminal tentacle. Scale bars: **(A)** = 50 µm; **(B,D,E)** = 60 µm; **(C)** = 30 µm.

**Figure 11:**
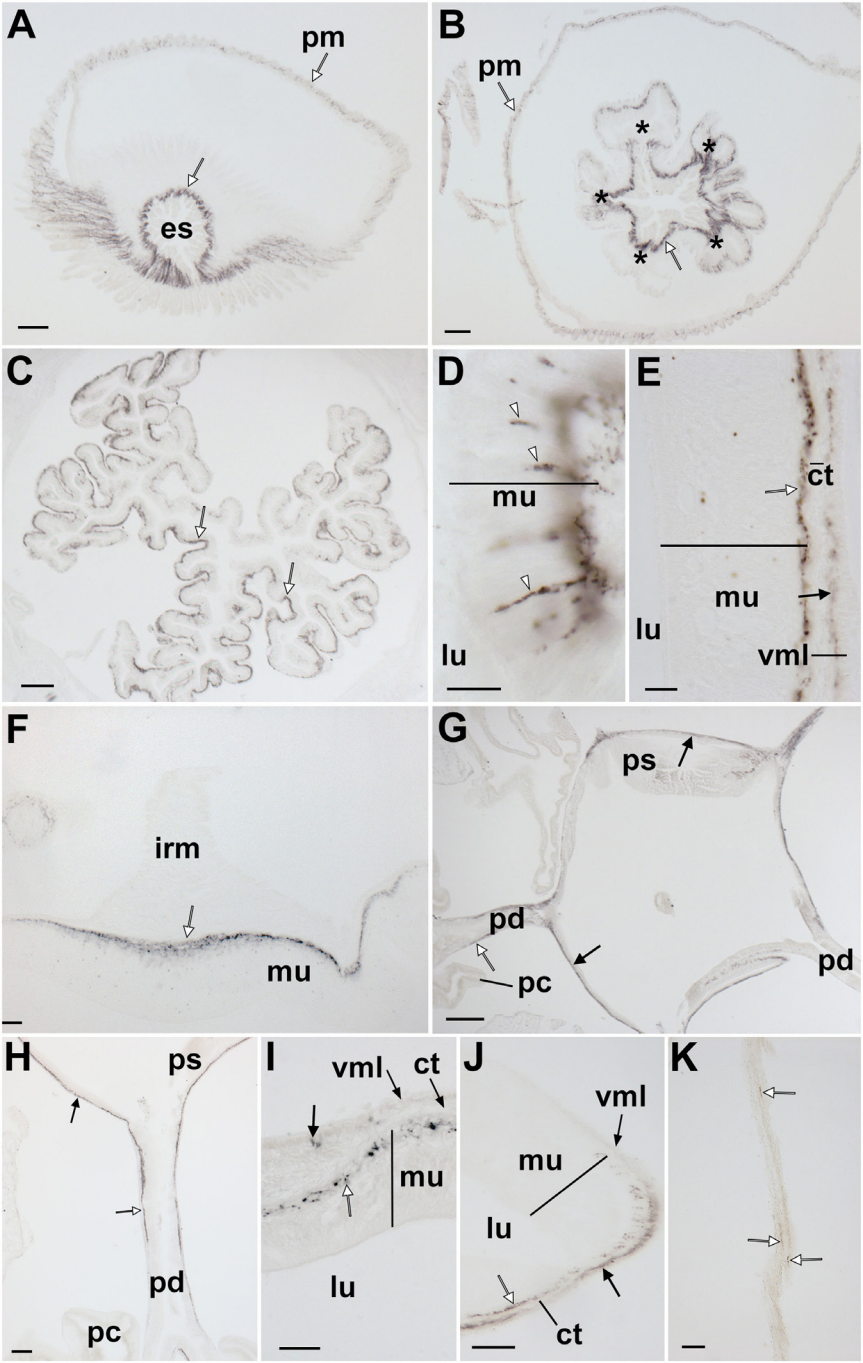
Localization of *Asterias rubens* gonadotropin-releasing hormone (ArGnRH) immunoreactivity in the digestive system of *A. rubens*. **(A)** Horizontal section through the central disk region near to the mouth, showing immunostaining in the peristomial membrane and esophagus. **(B)** Horizontal section through the central disk region showing immunostaining in the pentaradially symmetrical (asterisks) oral region of the cardiac stomach. **(C)** Horizontal section through the central disk showing immunostaining in the folded pouches of the cardiac stomach. **(D)** High magnification image of the cardiac stomach showing immunostained bipolar-shaped cells (white arrowheads) in the mucosal layer. **(E)** High magnification image of the cardiac stomach showing immunostaining in the basiepithelial nerve plexus (white arrow) and the visceral nerve plexus (black arrow) that is closely associated with the visceral muscle layer. **(F)** High magnification image showing immunostaining in a thickening of the basiepithelial nerve plexus that underlies a cardiac stomach intrinsic retractor muscle. **(G)** Horizontal section through the central disk showing immunostaining (white arrows) in the pyloric stomach and the pyloric ducts. **(H)** High magnification image showing immunostaining in a pyloric duct (white arrow) and in the pyloric stomach (black arrow). **(I)** High magnification image showing immunostaining in the basiepithelial nerve plexus of the mucosal layer and in a few processes associated with the visceral muscle layer. **(J)** High magnification image showing immunostaining in the basiepithelial nerve plexus (white arrow) and visceral nerve plexus (black arrow) on the oral side of a pyloric duct. **(K)** High magnification image showing immunostaining (white arrows) in a mesentery. Abbreviations: ct, collagenous tissue; es, esophagus; irm, instrinsic retractor muscle; lu, lumen; mu, mucosa, pc, pyloric caeca; pd, pyloric duct; pm, peristomial membrane; ps, pyloric stomach; vml, visceral muscle layer. Scale bars: **(A,B)** = 60 µm; **(C,G)** = 120 µm; **(D,E,I,J)** = 20 µm; **(F,H)** = 50 µm; **(K)** = 30 µm.

**Figure 12:**
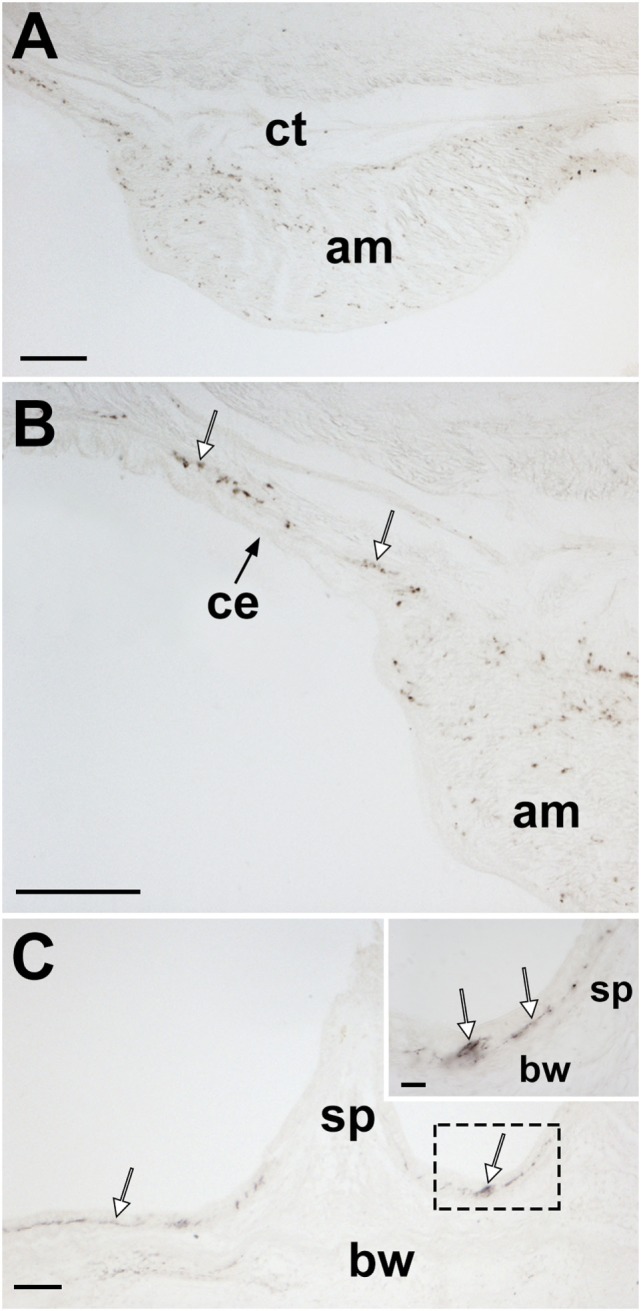
Localization of *Asterias rubens* gonadotropin-releasing hormone (ArGnRH) immunoreactivity in body wall-associated structures in *A. rubens*. **(A)** Immunostaining in a transverse section of the apical muscle. **(B)** Immunostained processes (white arrows) can be seen here in the basiepithelial plexus underlying the coelomic epithelium of the body wall and in the apical muscle. **(C)** Immunostained processes (white arrows) in the basiepithelial nerve plexus beneath the external epithelium of the body wall. The inset shows a higher magnification image of the boxed region. Abbreviations: am, apical muscle; bw, body wall; ce, coelomic epithelium; ct, collagenous tissue; sp, spine. Scale bars: **(A,B)** = 80 µm; **(C)** = 60 µm; [**(C)** inset] = 20 µm.

Detailed descriptions of the anatomy of the starfish *A. rubens* (Figures 1A,B) and other starfish species can be found in textbooks (43), reviews (44) and research articles (41, 45–48). However, to aid interpretation of images shown in Figures 2–6 (mRNA *in situ* hybridization) and Figures 7–12 (immunohistochemistry), in Figure 1 we show images of trichrome-stained sections of the arm (Figure 1C) and central disk (Figure 1D) in *A. rubens*, which were prepared using methods reported previously (41).

The main components of the nervous system in *A. rubens* are the radial nerve cords that extend along the oral (lower) side of each arm, with two rows of tube feet on either side of the radial nerve cord (Figure 1C). The radial nerve cords are linked by the circumoral nerve ring in the central disk (Figure 1D). Running parallel with the radial nerve cords are smaller marginal nerves, which are located lateral to the outer row of tube feet on each side of the arm (Figure 1C).

The digestive system of *A. rubens* comprises a mouth located on the underside of the central disk and surrounded by a contractile peristomial membrane, which is continuous aborally with a short tubular esophagus (Figure 1D). The stomach comprises two compartments: (1) the large and highly folded cardiac stomach, which is everted through the mouth during feeding (Figure 1D). (2) the smaller pyloric stomach (Figure 1D), which is linked *via* a short rectum to the anus located on the aboral surface of the central disk. Paired digestive glands or pyloric caeca are located in each arm (Figure 1C) and these are connected to the pyloric stomach by pyloric ducts (Figure 1D).

The body wall skeleton of *A. rubens* comprises calcite ossicles that are interconnected by muscles and collagenous tissue. The external surface of the body wall has a variety of appendages that include protective pincer-like pedicellariae and spines as well as thin-walled gas exchange organs known as papulae. A specialized tube foot-like organ located at the tips of each arm is the terminal tentacle, a sensory organ with a photosensitive and pigmented optic cushion located at its base. The internal surface of the body wall is lined by a coelomic epithelium, which is underlain by longitudinally and circularly orientated muscle layers. Along the midline of each arm the longitudinal muscle layer of the coelomic lining is thickened to form the apical muscle (Figure 1A), which facilitates flexion of the arm.

### Analysis of ArGnRHP Expression in *A. rubens* Using mRNA *In Situ* Hybridization

Experiments testing antisense probes on sections of arms from *A. rubens* revealed stained cells in the radial nerve cords (Figure 2A). Importantly, the specificity of staining with antisense probes was demonstrated by control experiments with sense probes, where no staining was observed (Figure 2A inset). Analysis of staining in the radial nerve cords revealed that ArGnRHP is expressed by cells in both the ectoneural and hyponeural regions. In the ectoneural region, stained cells are sparsely distributed throughout the subcuticular epithelial layer of the radial nerve cords, as seen in both transverse (Figure 2B) and parasagittal longitudinal (Figures 2C,D) sections of arms. In the hyponeural region, there are bilaterally symmetrical clusters of strongly stained cells (Figure 2B) and analysis of parasagittal longitudinal sections of arms revealed that these stained cell clusters are distributed along the length of the radial nerve cord, interrupted by regions without stained cells. Consistent with the pattern of expression in the radial nerve cords, ArGnRHP-expressing cells were revealed in both the ectoneural and hyponeural regions of the circumoral nerve ring (Figure 2E). Anatomically, the circumoral nerve ring corresponds to one half of the V-shaped radial nerve cord and accordingly only single clusters of stained cells are observed in the hyponeural region in transverse sections of the circumoral nerve ring. In the ectoneural region of the circumoral nerve ring, stained cells located in the subcuticular epithelial layer are largely grouped in a distinct band that is located approximately mid-way between the junction of the circumoral nerve ring with the peristomial membrane and the junction with perioral appendages (Figure 2E). ArGnRHP-expressing cells are also present in the marginal nerves (Figure 2F).

In the tube feet, ArGnRHP-expressing cells are located in a subepithelial position along the length of the tube foot stem (Figure 3A) and associated with the basal nerve ring (Figure 3B). At the tips of each arm ArGnRHP-expressing cells are present in the optic cushion (Figure 3C), the terminal tentacle (Figure 3D), flaps of tissue known as lateral lappets located adjacent to the terminal tentacle (Figure 3D), and the body wall epithelium surrounding the terminal tentacle (Figure 3D).

A very sparse population of cells expressing ArGnRHP is present in both the cardiac stomach (Figures 3E,F) and pyloric stomach (Figure 3G), with elongate stained cells located in the mucosal layer (Figure 3E) and roundish stained cells located in close association with the basiepithelial nerve plexus (Figure 3F). No ArGnRHP-expressing cells were observed in the pyloric ducts and the pyloric caeca, but stained cells were present in mesenteries that attach the pyloric caeca to the aboral body wall (Figure 3H).

### Analysis of ArCRZP Expression in *A. rubens* Using mRNA *In Situ* Hybridization

Experiments testing antisense probes on sections of arms from *A. rubens* revealed stained cells in the radial nerve cords (Figure 4A) and the specificity of staining with antisense probes was demonstrated by control experiments with sense probes, where no staining was observed (Figure 4A inset). Analysis of staining in the radial nerve cord revealed that ArCRZP is expressed by cells in the ectoneural region but not in the hyponeural region. The ArCRZP-expressing cells in the ectoneural region were sparsely distributed throughout the subcuticular epithelial layer both transversely (Figures 4A,B) and longitudinally (Figure 4C). Consistent with this pattern of expression in the radial nerve cords, ArCRZP-expressing cells were likewise observed only in the ectoneural region of the circumoral nerve ring. These cells were mostly distributed sparsely within the subcuticular epithelial layer (Figure 4D), but some cell clusters were also observed (Figure 4E).

Proximal to the outer rows of tube feet, ArCRZP-expressing cells were detected in the marginal nerves (Figure 5A). In the tube feet, ArCRZP-expressing cells were observed close to the junction between adjacent podia (Figure 5B). Stained cells were also sparsely distributed in a subepithelial location along the length of the tube foot stem and closely associated with the basal nerve ring (Figure 5C). Stained cells were observed in the external epithelium of the body wall and these cells were located at the junction between adjacent spines (Figure 5D). Stained cells were also associated with the apical muscle, with stained cells most prominent on the oral side of the muscle just beneath the coelomic epithelium. However, stained cells were also evident on the aboral side of the muscle close to the circular muscle layer (Figure 5E).

In the digestive system, ArCRZP expression was detected in the cardiac stomach, pyloric stomach and pyloric ducts (Figure 6). An extensive population of strongly stained cells was revealed in the pyloric stomach and the aboral part of the cardiac stomach and this was the most prominent expression of ArCRZP in the entire animal (Figure 6A). Stained cells in the cardiac stomach (Figure 6B) and the pyloric stomach (Figure 6C) included cells with an elongate shape located in the mucosal layer and roundish-shaped cells located close to the position of the basiepithelial nerve plexus. A similar pattern of expression was observed in the pyloric duct, but stained cells were only observed on the oral (lower) side of the pyloric duct (Figures 6D–F).

### Characterization of Antisera to ArGnRH and ArCRZ

#### ArGnRH Antiserum

Analysis of the ArGnRH antiserum (terminal bleed) using ELISA revealed the presence of antibodies to the antigen peptide. Furthermore, tests with an antiserum dilution series (10^−3^ to 10^−14^) were indicative of a high antibody titer, with the antigen clearly detected at dilutions in the range 10^−3^ to 10^−9^ (Figure S1A in Supplementary Material). However, preliminary immunohistochemical tests with diluted antiserum on sections of starfish arms revealed both ArGnRH-specific immunostaining and background immunostaining (data not shown). Therefore, ArGnRH-specific antibodies were affinity-purified from the antiserum using the AminoLink^®^ Plus Immobilization Kit (Thermo Scientific). Analysis of column eluates using ELISA revealed the presence of antibodies to ArGnRH in the triethylamine eluate but not in the glycine eluate (Figure S1B in Supplementary Material). Therefore, the triethylamine-eluted antibodies to ArGnRH were used for immunohistochemical analysis of ArGnRH expression in *A. rubens* (see below).

#### ArCRZ Antisera

Enzyme-linked immunosorbent assay analysis of antiserum (terminal bleed) from a rabbit immunized with an antigen corresponding to the C-terminal region of ArCRZ (KYGQNRWKAG-NH_2_) revealed no evidence of antibodies to the antigen (Figure S1C in Supplementary Material). Therefore, a second rabbit antiserum was generated using an antigen comprising an analog of the C-terminal region of ArCRZ with the lysine residue corresponding to the thirteenth residue replaced with arginine (ArCRZ[Arg^13^]; see methods for the rationale). ELISA analysis of the ArCRZ[Arg^13^] antiserum revealed the presence of antibodies to the antigen peptide, but only with high concentrations of antiserum (Figure S1C in Supplementary Material). The terminal antiserum was subject to affinity-purification, but antibodies to the antigen peptide were not detected by ELISA (Figure S1D in Supplementary Material). Antiserum collected at day 38 was also subject to affinity purification and ELISA revealed antibodies in the triethylamine eluate, but only with the highest antibody concentration tested (Figure S1D in Supplementary Material). In conclusion, we were unsuccessful in generating antibodies to ArCRZ that could be used for immunohistochemical localization of this peptide in *A. rubens*.

### Analysis of ArGnRH Expression in *A. rubens* Using Immunohistochemistry

#### Radial Nerve Cords, Circumoral Nerve Ring, and Marginal Nerves

ArGnRH-immunoreactivity (ir) was observed in all the major components of the nervous system of *A. rubens*, including the radial nerve cords, the circumoral nerve ring and the marginal nerves (Figures 7 and 8). Furthermore, the specificity of this immunostaining was confirmed by control experiments where no immunostaining was observed in sections incubated with primary antibodies preabsorbed with ArGnRH peptide (Figure 8A inset). In the radial nerve cord, ArGnRH-ir is present in both the hyponeural and ectoneural regions (Figure 8A).

In the hyponeural region of the radial nerve cords, ArGnRH-ir cells are grouped in bilaterally symmetrical clusters that are located, as viewed in transverse sections (Figures 8A,B), in its lowermost (oral) zone proximal to the junction between the radial nerve cord and the radial heamal strand. In longitudinal sections it can be seen that the ArGnRH-ir cell clusters correspond with the segmental structure of the ambulacrum, with a ArGnRH-ir cell cluster corresponding with each tube foot (Figures 7A,C). High magnification images of the hyponeural ArGnRH-ir cell bodies show that they are monopolar cells with associated axonal processes (Figure 8C). Immunostained processes in the hyponeural region can be seen projecting from the radial nerve cord around the lateral wall of the radial perihemal canal (Figure 8E).

In the ectoneural region of the radial nerve cords, ArGnRH-ir is present in bipolar-shaped cells located in the subcuticular epithelium, with immunostained processes extending into the underlying neuropile (Figure 8D). Stained cells are most prominent in the lower two-thirds of its V-shaped structure, with lateral regions proximal to the tube feet largely void of stained cells (Figure 8A). Intense immunostaining is present in the underlying neuropile of the ectoneural region and interestingly there are distinct compartments that are more intensely stained than others. Thus, in the lower (oral) half of the radial nerve cords (as seen in transverse sections) there is an intensely stained zone(s) containing a dense meshwork of labeled fibers. In contrast, in the upper and lateral regions of the radial nerve cord, the intensity and density of staining is much lighter. Thus, the pattern of staining in the ectoneural neuropile region corresponds with the distribution of stained ectoneural cell bodies (see above). The compartmentalization of intensely stained and less intensely stained regions of the ectoneural neuropile can also clearly be seen in longitudinal sections, both in the parasagittal plane (Figure 7A) and in the horizontal plane (Figure 7B). Immunostained cells and processes are also present in the marginal nerves, thickenings of the basiepithelial plexus that run along length of the arms adjacent to the outer row of tube feet (Figures 7D and 8F).

The pattern of immunostaining observed in the circumoral nerve ring was consistent with that observed in the radial nerve cords. Thus, stained cells are present in both the hyponeural and ectoneural regions (Figures 9A,B). The stained cells in the hyponeural region are monopolar cells (Figure 9C), whereas stained cells in the ectoneural epithelium are bipolar cells with a short bulbous projection located basally that is proximal to the cuticular layer and a long apical axonal process that projects into the neuropile layer (Figure 9D). As in the radial nerve cords, immunostained cells are not evenly distributed throughout the ectoneural epithelium, but are concentrated in a region corresponding to the medial zone of the circumoral nerve ring when viewed in transverse section (Figure 9A). The distribution and intensity of immunostaining in the underlying ectoneural neuropile of the circumoral nerve ring corresponds with the distribution of stained cells (Figures 9A,B). Immunostained processes can be seen projecting from the ectoneural neuropile into the basiepithelial plexus of the peristomial membrane (Figure 9A). Similarly, immunostained processes in the lateral region of the ectoneural neuropile can be seen projecting into the basiepithelial nerve plexus of the adjacent body wall (Figure 9A).

#### Tube Feet and Terminal Tentacle

In tube feet, immunoreactivity is present in fibers located within the basiepithelial nerve plexus along the stem of the podium (Figure 10A) and in the basal nerve ring of the tube foot sucker (Figures 10A,B). Immunostained cells and/or processes can also be seen at the junction between the tube foot stem and the radial nerve cord (Figure 10C) and at the junction between adjacent tube feet (Figure 10D). The optic cushion located at the base of the terminal tentacle contains immunoreactive cells and processes (Figure 10E). Furthermore, immunostained cells are also present in the external epithelial layer surrounding the terminal tentacle and immunostained processes are present in the underlying basiepithelial nerve plexus (Figure 10E).

#### Digestive System

ArGnRH-ir was revealed in several regions of the digestive system, including the peristomial membrane (Figure 11A), the esophagus (Figures 11A,B), the cardiac stomach (Figures 11C–F), the pyloric stomach (Figures 11G–I), and the pyloric ducts (Figure 11J). The majority of the immunostaining is localized to nerve fibers in the basiepithelial nerve plexus in each of these regions of the digestive system (Figures 11A–C). Furthermore, in regions of the cardiac stomach where the basiepithelial nerve plexus is thickened, such as sites of insertion of the intrinsic retractor strands, there is a corresponding thickening of the plexus of ArGnRH-immunoreactive fibers (Figure 11F). Immunostained bipolar-shaped cells located in the mucosal layer of the digestive system were also observed; for example, in the cardiac stomach (Figure 11D). In the pyloric stomach, immunostained processes can be seen in both the basiepithelial nerve plexus and the nerve plexus associated with the visceral muscle layer (Figure 11I). Analysis of transverse sections of the pyloric ducts revealed that an immunostained fiber plexus is located on the oral (lower) side (Figure 11J). Although the pyloric caeca were largely void of immunostaining (Figure 11G), immunoreactive fibers were present in the mesenteries that attach the pyloric caeca to the aboral body wall (Figure 11K).

#### Body Wall-Associated Structures

Immunostained processes are present in the longitudinally orientated muscle layer of the apical muscle and in the basiepithelial plexus underlying the coelomic epithelium of the aboral body wall (Figures 12A,B). Immunostained processes are also observed in the basiepithelial nerve plexus beneath the external epithelium of the body wall (Figure 12C).

### ArGnRH and ArCRZ Cause Contraction of Cardiac Stomach, Apical Muscle, and Tube Foot Preparations from *A. rubens*

Analysis of the *in vitro* pharmacological effects of ArGnRH and ArCRZ revealed that both peptides cause contraction of cardiac stomach, tube foot, and apical muscle preparations, but with differing potency/efficacy.

**Figure 13:**
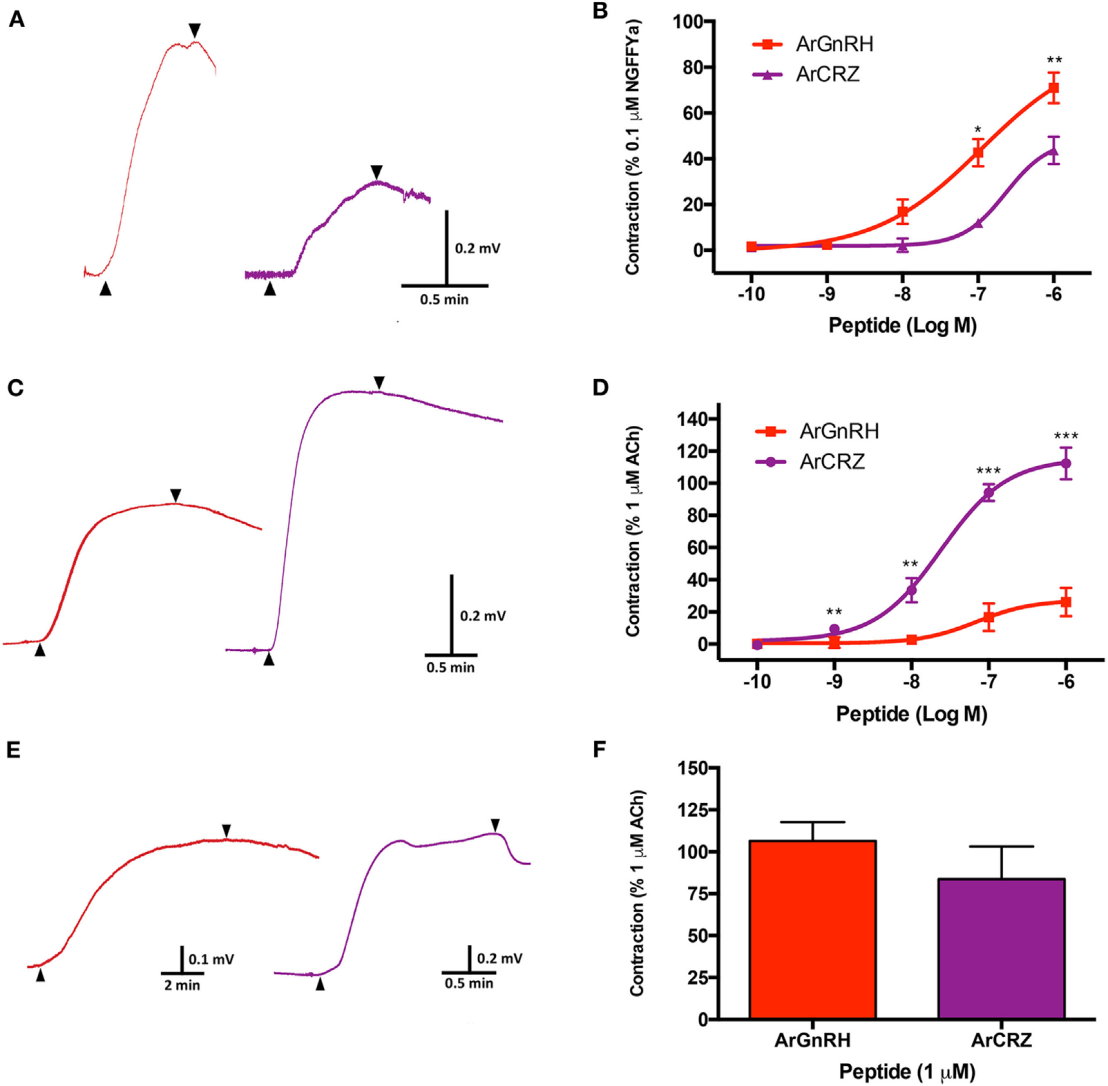
Comparison of the pharmacological effects of *Asterias rubens* gonadotropin-releasing hormone (ArGnRH) and *A. rubens* corazonin (ArCRZ) on cardiac stomach, apical muscle, and tube foot preparations from the starfish *A. rubens*. **(A)** Representative recordings from a single cardiac stomach preparation showing the contraction-inducing effects of ArGnRH (red line) and ArCRZ (purple line) at 10^−6^ M. Peptide application is labeled with an upward pointing arrowhead, washing of the preparation is labeled with a downward pointing arrowhead. **(B)** Graphs showing the dose-dependent effects of ArGnRH (red) and ArCRZ (purple) on cardiac stomach, expressed as mean percentages (±SEM) of the contraction induced by 10^−7^ M NGFFYamide. Data were collected from at least five independent experiments. The effect of ArGnRH was significantly larger than the effect of ArCRZ when tested at 10^−7^ and 10^−6^ M. **(C)** Representative recordings from a single apical muscle preparation showing the contraction-inducing effects of ArGnRH (red line) and ArCRZ (purple line) at 10^−6^ M. Peptide application is labeled with an upward pointing arrowhead, washing of the preparation is labeled with a downward pointing arrowhead. **(D)** Graphs showing the dose-dependent effects of ArGnRH (red) and ArCRZ (purple) on apical muscle preparations, expressed as mean percentages (±SEM) of the contraction induced by 10^−6^ M acetylcholine. Data were collected from at least five independent experiments. The effect of ArCRZ was significantly larger than the effect of ArGnRH when tested at 10^−9^, 10^−8^, 10^−7^, and 10^−6^ M. **(E)** Representative recordings from a single tube foot preparation showing the contraction-inducing effects of ArGnRH (red line) and ArCRZ (purple line) at 10^−6^ M. Peptide application is labeled with an upward pointing arrowhead, washing of the preparation is labeled with a downward pointing arrowhead. **(F)** Comparison of the effects of ArGnRH and ArCRZ on tube foot preparations, expressed as mean percentages (±SEM) of the contraction induced by 10^−6^ M acetylcholine. The data were collected from at least three independent experiments. Student’s *t*-tests were performed to determine whether there were significant differences in the effects of ArGnRH and ArCRZ when tested at the same concentration (***p < 0.001, **p < 0.01; *p < 0.05).

ArGnRH was more potent and effective than ArCRZ as a contractant of cardiac stomach preparations (Figures 13A,B). Thus, ArGnRH caused dose-dependent contraction of cardiac stomach preparations at concentrations between 10^−6^ and 10^−8^ M (Figure 13B). The *E*_max_ for ArGnRH at a concentration of 10^−6^ M was 71 ± 6.7% in comparison with the effect of NGFFYamide at 10^−7^ M (defined as 100% contraction). ArCRZ caused contraction of cardiac stomach preparations at 10^−6^ and 10^−7^ M and the *E*_max_ for ArCRZ at 10^−6^ M was only 43.7 ± 5.9% when compared with NGFFYamide (10^−7^ M). Accordingly, comparison of the effects of ArGnRH and ArCRZ when tested at 10^−6^ and 10^−7^ M revealed that the effect of ArGnRH was significantly larger than the effect of ArCRZ, supported by *p* values (Student’s *t*-test) of 0.012 and 0.002, respectively.

ArCRZ was more potent and effective than ArGnRH as a contractant of apical muscle preparations (Figures 13C,D). Thus, ArCRZ induced dose-dependent contraction of apical muscle preparations at concentrations between 10^−6^ and 10^−9^ M (Figure 13D). The *E*_max_ for ArCRZ at a concentration of 10^−6^ M was 112.4 ± 10% in comparison with the effect of ACh at 10^−6^ M (defined as 100% contraction) (Figure 13D). ArGnRH caused contraction of apical muscle preparations at 10^−6^ and 10^−7^ M but the *E*_max_ for ArGnRH at 10^−6^ M was only 26.3 ± 4% when compared with ACh (10^−6^ M). Accordingly, comparison of the effects of ArCRZ and ArGnRH when tested at 10^−6^ and 10^−7^ M revealed that the effect of ArCRZ was significantly larger than the effect of ArGnRH, supported by *p* values (Student’s *t*-test) of 3.943 × 10^−5^ and 6.000 × 10^−7^, respectively.

Both ArGnRH and ArCRZ caused contraction of tube foot preparations at 10^−6^ M (Figures 13E,F), but effects were not observed when tested at lower concentrations (10^−7^ to 10^−10^ M). In comparison with the effect of ACh at 10^−6^ M, the *E*_max_ for ArGnRH was 106.5 ± 11.21% whereas the *E*_max_ for ArCRZ was 83.7 ± 19.5% (Figure 13F). However, statistical analysis revealed that there was no significant difference in the effects of ArGnRH and ArCRZ at 10^−6^ M (*p* = 0.328; Student’s *t*-test).

### ArGnRH and ArCRZ Do Not Induce Cardiac Stomach Retraction in *A. rubens*

In 10 specimens of *A. rubens* that were injected with ArGnRH, no cardiac stomach retraction was observed in the 5 min observation period, whereas NGFFYamide induced cardiac stomach retraction in all 10 of these animals (but with variability in the rate and extent of the retraction; data not shown). Likewise, cardiac stomach retraction was not observed in any of the 10 animals injected with ArCRZ, whereas NGFFYamide induced cardiac stomach retraction in all 10 of these animals (but with variability in the rate and extent of the retraction; data not show).

## Discussion

Previously, we have reported the discovery of distinct GnRH-type and CRZ-type signaling systems in the starfish *A. rubens* (25). Here, we report a detailed analysis of the expression patterns and pharmacological activities of the GnRH-type neuropeptide ArGnRH and the CRZ-type neuropeptide ArCRZ in *A. rubens*. This is the first study to functionally characterize GnRH-type and CRZ-type neuropeptides in an echinoderm.

Use of mRNA *in situ* hybridization methods enabled anatomical visualization of transcripts encoding the ArGnRHP and ArCRZP. Both precursors are widely expressed, including cells located in the radial nerve cords, circumoral nerve ring, tube feet and digestive system. However, as discussed in more detail below, differences in the patterns of expression of the two precursors were observed when examined in detail in specific parts of the body. Generation of antibodies to ArGnRH enabled detailed immunohistochemical analysis of the distribution of cells and axonal processes containing this neuropeptide, which facilitated interpretation of the physiological relevance of the pharmacological actions of ArGnRH, as discussed below. Our efforts to generate antibodies to ArCRZ were unsuccessful, in spite of testing two different C-terminal peptide antigens. We conclude, therefore, that the C-terminal region of ArCRZ may exhibit low antigenicity.

Analysis of the *in vitro* pharmacological effects of ArGnRH and ArCRZ revealed that both peptides cause contraction of cardiac stomach, tube feet and apical muscle preparations from *A. rubens*. However, consistent with differences in the expression patterns of the ArGnRHP and ArCRZP, there were organ-specific differences in the effectiveness/potency of ArGnRH and ArCRZ as muscle contractants, as discussed in more detail below.

### Functional Interpretation of the Anatomy and Pharmacological Actions of ArGnRH and ArCRZ in *A. rubens*

#### Radial Nerve Cords, Circumoral Nerve Ring, and Marginal Nerves

Based upon data obtained using mRNA *in situ* hybridization it is clear that both ArGnRHP and ArCRZP are expressed widely in the nervous system of the *A. rubens*, including cells in the radial nerve cords, the circumoral nerve ring and the marginal nerves. However, a major difference in the expression patterns of ArGnRHP and ArCRZP is that ArGnRHP is expressed in both the ectoneural and hyponeural regions of the radial nerve cords and circumoral nerve ring, whereas ArCRZP-expressing cells are only detected in the ectoneural region. The marginal nerve comprises only ectoneural tissue and consistent with the expression pattern in the radial nerve cords and circumoral nerve ring, both ArGnRHP- and ArCRZP-expressing cells are detected in the marginal nerve. It remains to be determined, however, whether or not ArGnRHP and ArCRZP are coexpressed by cells in ectoneural regions of the nervous system.

Differences in the expression patterns of ArGnRHP and ArCRZP can be interpreted based on what is known about the functions of the ectoneural and hyponeural regions of the radial nerve cords and circumoral nerve ring. The ectoneural region is thought to comprise sensory neurons, interneurons, and motor neurons, whereas the hyponeural region only contains motor neurons (49–51). Between the ectoneural and hyponeural regions of the radial nerve cords and circumoral nerve ring is a thin layer of collagenous tissue and it has been proposed that because of the presence of this layer there are no direct anatomical connections between ectoneural and hyponeural neurons (49). However, recent detailed anatomical analysis of echinoderm nervous systems indicates that the ectoneural and hyponeural components are anatomically and functionally interconnected (52–54). Interestingly, our immunohistochemical analysis of ArGnRH expression in the radial nerve cords revealed that hyponeural ArGnRH-immunoreactive neurons are proximal to a region of the ectoneural neuropile that contains ArGnRH-immunoreactive fibers (see Figure 8C). At the light microscopic level, we observed no evidence of immunostained fibers crossing the collagenous tissue layer, but we cannot rule out the possibility that this occurs. In the lateral regions of the radial nerve cord it was, however, possible to see the projection pathway of the axonal processes of hyponeural neurons around the lateral walls of the peri-hemal canals, but it was not possible to trace these fibers any further. Therefore, it is not known what are the postsynaptic targets of the hyponeural ArGnRH-immunoreactive neurons. In the lateral regions of the nerve cords, the immunostained fibers in the ectoneural region of the radial nerve cords and the circumoral nerve ring can clearly be seen to be projecting into the basiepithelial nerve plexus of adjacent organs/tissues. Thus, in Figure 9A ectoneural immunoreactive fibers in the circumoral nerve ring can be seen projecting into the basiepithelial plexus of the body wall and peristomial membrane, while in Figure 10C ectoneural immunostained processes in the radial nerve cord can be seen to be contiguous with the basiepithelial plexus of an adjacent tube foot. Therefore, it is likely that ArGnRH released from these ectoneural fibers regulates the activity of the associated peripheral tissues/organs and evidence in support of this hypothesis is presented below with specific reference to tube feet.

#### Tube Feet and the Terminal Tentacle

Analysis of ArGnRHP and ArCRZP expression in tube feet using mRNA *in situ* hybridization revealed cells expressing these precursors at the junction between adjacent tube feet (ArCRZP; Figure 5B) and in a basiepithelial position along the length of the tube foot stem (ArGnRHP; Figure 3A). ArGnRHP- and ArCRZP-expressing cells are also present near the tube foot sucker where they appear to be closely associated with the basal nerve ring (Figures 3B and 5C). Consistent with this pattern of expression for ArGnRHP, immunohistochemical localization of ArGnRH revealed immunostained fibers in the basiepithelial plexus of the tube foot stem and in the tube foot basal nerve ring (Figures 10A,B). Collectively, these anatomical data indicate that both ArGnRH and ArCRZ may have physiological roles in regulation of tube foot activity. Accordingly, *in vitro* pharmacological experiments revealed that both ArGnRH and ArCRZ cause contraction of tube feet, albeit only at a relatively high concentration (10^−6^ M; Figures 13E,F). This low potency may reflect poor penetration of these peptides into the muscle layer of tube foot preparations *in vitro*. Similar findings have been reported previously when testing neuropeptides that cause relaxation of tube feet—the SALMFamides S1 and S2—where effects were also only observed with high peptide concentrations (10^−5^ M) (35). Efforts to improve peptide penetration were made in the previous study (35) and in this study by partially stripping the external epithelial layer of tube foot preparations. However, this may have limited effectiveness based on the relatively low potency of ArGnRH and ArCRZ on the one hand and S1 and S2 on the other hand as tube foot contractants and relaxants, respectively.

The presence of ArGnRHP and ArCRZP expressing cells and ArGnRH-immunoreactivity associated with the basal nerve ring, may be indicative of a role in neural regulation of the tube foot sucker. One important function of the tube foot sucker is to secret adhesive proteins, which enables tube feet to attach or detach from the substratum during locomotor activity (55). Interestingly, the expression ArGnRHP in the larval (brachiolaria) nervous system has been investigated recently and found to be localized in the basiepithelial nerve plexus under the adhesive disk (40). When starfish larvae undergo settlement prior to metamorphosis the adhesive disk mediates permanent attachment to a substratum (56). There may be similarities in the mechanisms controlling secretion of adhesive proteins from tube feet suckers and the larval adhesive disk and the associated expression of ArGnRH in both systems could be evidence of this.

At the tips of each arm in starfish is a tube foot-like organ known as the terminal tentacle, which is a sensory organ that has a pigmented optic cushion at its base (57). No ArCRZP expression was detected in the terminal tentacle or optic cushion, but ArGnRHP-expressing cells and ArGnRH-immunoreactive fibers are present in the terminal tentacle, lateral lappets, and optic cushion (Figures 3C,D and 10E). Immunostained fibers are also present in the basiepithelial nerve plexus of the body wall surrounding the terminal tentacle. The functional significance of the expression of ArGnRH in the terminal tentacle and associated structures remains to be determined. However, by analogy with its contracting effect on tube feet, ArGnRH may likewise cause retraction of the highly extendable terminal tentacles.

#### Digestive System

Both ArGnRHP and ArCRZP are widely expressed in the digestive system of *A. rubens*. Thus, ArGnRHP-expressing cells and ArGnRH-immunoreactive fibers are present in the peristomial membrane, esophagus, cardiac stomach, pyloric stomach, and pyloric caeca, while ArCRZP-expressing cells are present in the cardiac stomach, pyloric stomach and pyloric duct. *In vitro* pharmacological tests with synthetic ArGnRH and ArCRZ revealed that both neuropeptides cause dose-dependent contraction of cardiac stomach preparations (Figure 13B), but with ArGnRH being more potent and effective than ArCRZ. A previous study has revealed that the starfish neuropeptide NGFFYamide, which is orthologous to neuropeptide-S in vertebrates and crustacean cardioactive peptide in protostomian invertebrates (37), also causes contraction of *A. rubens* cardiac stomach preparations *in vitro* (38). Furthermore, administration of NGFFYamide *in vivo* triggers retraction of the everted cardiac stomach. Comparison of the effectiveness of NGFFYamide, ArGnRH and ArCRZ as cardiac stomach contractants when tested at 0.1 µM *in vitro* reveals that NGFFYamide is most effective, with the effect of ArGnRH and ArCRZ 42.63 ± 6.01 and 11.92 ± 2.18% of NGFFYamide-induced contraction, respectively (Figure 13B). These data indicate that ArGnRH and ArCRZ may be less important than NGFFYamide in neural regulation of cardiac stomach retraction in *A. rubens*. To address this issue here, we tested both ArGnRH and ArCRZ *in vivo* using a method similar to that used previously with NGFFYamide (38) but neither ArGnRH nor ArCRZ triggered cardiac stomach retraction in 10 animals tested.

While the effects of ArGnRH and ArCRZ as cardiac stomach contractants *in vitro* may be more modest than those of NGFFYamide, it is nevertheless of interest to explore relationships between the anatomical patterns of expression of ArGnRHP, ArGnRH and ArCRZP and the pharmacological effects of ArGnRH and ArCRZ *in vitro*. Analysis of the expression ArGnRHP in the digestive system using mRNA *in situ* hybridization revealed a very sparse population of stained cells in the cardiac stomach and pyloric stomach, which included bipolar-shaped cells in the mucosal epithelium and roundish-shaped cells closely associated with the basiepithelial nerve plexus. However, immunohistochemcial analysis revealed that the relatively sparse population of ArGnRHP-expressing cells gives rise to an extensive network of immunostained fibers in the basiepithelial nerve plexus of the cardiac stomach and pyloric stomach. Furthermore, immunohistochemistry also revealed immunostaining in the nerve plexus associated with visceral muscle layer (Figures 11E,I), which is separated from the basiepithelial nerve plexus by a thin layer of collagenous tissue (48). Importantly, the presence of ArGnRH in fibers closely associated with visceral muscle layer of the cardiac stomach and pyloric stomach is consistent with the *in vitro* effect of ArGnRH in causing contraction of the cardiac stomach. Furthermore, ArGnRH released by fibers in the basiepithelial nerve plexus may also affect the contractile state of the visceral muscle layer if peptide molecules diffuse across the intervening collagenous layer.

While we have obtained evidence that ArGnRH regulates the contractility of the visceral muscle layer in the cardiac stomach of *A. rubens*, it would be simplistic to conclude that this is its only role in the digestive system. For example, ArGnRH immunoreactive fibers are also present in the pyloric ducts, regions of the digestive system than enable ciliary-mediated movement of gut contents between the pyloric stomach and the pyloric caeca (46, 58–60) (Figure 11J). Therefore, ArGnRH may also be involved in regulation of ciliary beating in the pyloric ducts and in other regions of the digestive system. Such a role would be consistent with actions of neuropeptides as regulators of ciliary activity in the adult and larval forms of other animals (61–63).

Analysis of the expression of ArCRZP in the digestive system of *A. rubens* using mRNA *in situ* hybridization revealed an extensive population of stained cells in the aboral region of the cardiac stomach and in the pyloric stomach. Stained cells were revealed in the mucosal layer but we did not observe expression in association with the visceral muscle layer, which may correlate physiologically with the relatively modest effect of ArCRZ on cardiac stomach by comparison with ArGnRH. Interestingly, stained cells were sparse or absent in the oral region of the cardiac stomach, which is the region that is everted through the mouth during feeding. This pattern of expression may also correlate physiologically with the relatively modest effects of ArCRZ in causing contraction of the cardiac stomach because it is the highly folded oral region of the cardiac stomach that forms the bulk of the organ when it is set up as an *in vitro* preparation [see Figure 1 of Ref. (32)]. Furthermore, as discussed above with respect to ArGnRH, the physiological roles of ArCRZ may extend beyond regulation of myoactivity and, as with ArGnRH, the presence of ArCRZ-expressing cells in regions of the digestive system such as the pyloric ducts may be indicative of other functions (e.g., regulation of ciliary activity).

#### The Apical Muscle and Other Body Wall-Associated Organs

Analysis of ArGnRHP and ArCRZP expression in the body wall using mRNA *in situ* hybridization revealed cells expressing ArCRZP in the external epithelium and the coelomic epithelium of the body wall (Figures 5D,E) while ArGnRHP expression was not detected in these epithelia. However, the latter finding may reflect insensitivity of mRNA *in situ* hybridization in detecting ArGnRH transcripts because immunohistochemical analysis revealed immunostained processes in the basiepithelial plexi of the external epithelium (Figure 12C) and the coelomic epithelium (Figure 12B).

The apical muscle is a thickening of the longitudinally orientated muscle layer of the coelomic lining that runs along the midline of the aboral body wall in each arm of *A. rubens*. ArCRZP-expressing cells were detected in coelomic epithelium of the apical muscle and in the circular muscle layer beneath the apical muscle (Figure 5E). As in other regions of the body wall (see above), ArGnRHP expression was not detected in the apical muscle but immunohistochemical analysis revealed immunostained axon profiles among the longitudinally orientated muscle fibers of the apical muscle (Figures 12A,B). Thus, evidence that both ArCRZ and ArGnRH may be involved in regulation of apical muscle activity was obtained from molecular anatomical analysis. Accordingly, *in vitro* pharmacological tests revealed that both ArCRZ and ArGnRH cause dose-dependent contraction of apical muscle preparations (Figure 13D). However, ArCRZ was more potent and effective than ArGnRH as a contractant of the apical muscle, the opposite of what was found with cardiac stomach preparations *in vitro* (see above and Figure 13B). Interestingly, when tested at 1 µM ArCRZ was equally as or slightly more effective than 1 µM acetylcholine as a contractant of the apical muscle. Acetylcholine is known to act as neuromuscular transmitter in echinoderms (64, 65) and therefore the comparable activity of ArCRZ as a apical muscle contractant is noteworthy. We conclude, therefore, that ArCRZ, and perhaps to lesser extent ArGnRH, may be participate in neural mechanisms controlling apical muscle contraction; for example, during arm flexion when starfish exhibit changes in posture associated with feeding or spawning behavior (66, 67).

### Evolution and Comparative Physiology GnRH/CRZ-Type Neuropeptides in the Bilateria

As highlighted in the Section “Introduction,” the discovery of ArGnRH and ArCRZ as respective ligands for GnRH-type and CRZ-type receptors in the starfish *A. rubens* provided an important new insight into the evolution of GnRH/CRZ-type neuropeptide signaling (25). It demonstrated that the evolutionary origin of these paralogous neuropeptide signaling pathways can be traced back to the common ancestor of protostomes and deuterostomes, but with subsequent loss in some lineages (e.g., loss of CRZ signaling in vertebrates, nematodes, and urochordates) and duplication of the GnRH-signaling system in arthropods to give rise to the paralogous AKH-type and ACP-type signaling pathways. Informed by these findings, we have reviewed published research on GnRH/CRZ-type neuropeptide signaling and have proposed a standardized nomenclature wherein peptides are either named “GnRH” or “corazonin,” with the exception of the paralogous “AKH/RPCH”-type and “ACP”-type neuropeptides that arose by gene duplication in the arthropod lineage (26). Reviewing published literature in this context reveals that some neuropeptides hitherto referred to as GnRH or invertebrate-GnRH are in fact ligands for CRZ-type receptors and therefore we have proposed that these should instead be referred to as CRZs. Furthermore, GnRH-type peptides in non-arthropodan protostomian invertebrates have often been named “AKH,” whereas we have proposed that a more suitable name for these peptides would be GnRH because AKH is a unique product of gene duplication in the arthropod lineage (26). With these nomenclature issues in mind, here we present a selective review of what is known about the physiological roles of GnRH/CRZ-type neuropeptides in bilaterian animals so that the findings reported here from *A. rubens* can be considered in a broader comparative and evolutionary context.

As the prototype of the GnRH/CRZ neuropeptide family, the physiological role of GnRH in mammals and other vertebrates as regulator of pituitary release of the gonadotropic hormones luteinizing hormone and follicle stimulating hormone may have influenced expectations of the physiological roles of homologous peptides in other taxa. Interestingly, evidence of roles of GnRH/CRZ-type neuropeptides in regulation of reproductive physiology have been obtained in some invertebrates, including the urochordate *C. intestinalis* (6) and several molluscan species (68). In this context it is interesting that an ortholog of ArGnRH was found not to induce release of the gonadotropin relaxin-like gonad-stimulating peptide (RGP) in the starfish *Patiria pectinifera* when tested *in vitro* (69). Furthermore, we have observed that ArGnRH does not induce spawning of gonadal fragments from *A. rubens in vitro* (M. Mita, S. Tian, M.R. Elphick, unpublished data) when tested in parallel with RGP, which does induce spawning (39). Therefore, based on the data currently available it appears that GnRH-type neuropeptides may not be involved in regulation of reproductive physiology in starfish.

Much of what we know about the physiological roles of GnRH/CRZ-type neuropeptides in invertebrates is based on findings from insects and to a lesser extent other arthropods. The first GnRH-type peptide to be identified in an invertebrate was the crustacean hormone RPCH (13), which causes alterations in body coloration due to pigment migration in chromatophores (13, 70). Subsequently, an RPCH-like peptide that triggers lipid mobilization was identified in the locust *Schistocerca gregaria*, and hence it was named AKH (11, 12, 14). However, more recent studies have revealed that AKH also has a variety of other physiological roles in insects (26). Less is known about the physiological roles of ACP, the paralog of AKH in arthropods, and this in part due to the fact that it was discovered more recently and because ACP signaling has been lost in the principal arthropod model system*—Drosophila*. Nevertheless, the data that are available are indicative of physiological roles distinct from those of AKH (71, 72).

Corazonin was first identified in the cockroach *Periplaneta americana* on account of its cardioacceleratory effect (15) and then from locusts using an immunoassay (73). Interestingly, CRZ was also identified independently as a peptide that triggers dark pigmentation in locusts (74). Furthermore, as with AKH, more recent studies have revealed that CRZ also has a variety of other physiological roles in insects (26).

Comparison of the physiological roles of AKH/ACP-type and CRZ-type signaling in arthropods reveals some similarities, although this varies between different lineages. For example, both AKH and CRZ affect responses to stress in *Drosophila* (75–78), while in the crayfish *Procambarus clarkii* both CRZ and RPCH affect pigment migration (79, 80), Presumably these and other similarities in the physiological roles of AKH/ACP and CRZ signaling in arthropods (26) are a reflection of their common evolutionary origin, while differences may reflect neofunctionalization or subfunctionalization.

A similar mix of similarities and differences in the physiological roles of GnRH/CRZ-type signaling have been revealed by an elegant series of experimental studies on the marine mollusk *Aplysia californica*. However, interpretation of the published data is complicated by nomenclature issues. Two GnRH/CRZ-type neuropeptide signaling systems have been identified in *Aplysia*, one referred to as GnRH and the other referred to as AKH (81–83). However, *Aplysia* “GnRH” is actually the ligand for a CRZ-type receptor (83) and hence we have proposed that this peptide should be named *Aplysia californica* corazonin (AcCRZ), and *Aplysia* “AKH” should be renamed *Aplysia californica* GnRH (AcGnRH) (26). Employing use of the proposed revised nomenclature, below we summarize what is known about the physiological roles of GnRH/CRZ-type neuropeptides in *Aplysia*.

Neurons expressing the AcGnRH precursor are located in the abdominal, cerebral, and pleural ganglia, but the processes of these neurons are present in all ganglia, indicating potential roles in regulation of a variety of physiological processes. Injection of AcGnRH inhibits feeding, reduces body mass, increases defecation, and reduces gonadal mass and oocyte diameter (82). Analysis of the expression of the AcCRZP revealed expression in the pedal, cerebral and abdominal ganglia (84). Furthermore, *in vivo* pharmacological experiments revealed that AcCRZ triggers parapodial opening, inhibition of feeding, and promotion of substrate attachment but has no effect on oocyte growth, ovotestis mass, reproductive tract mass, egg-laying, egg-laying hormone accumulation and secretion, and penile eversion (81). Thus, there are distinct differences in the physiological roles of GnRH-type and CRZ-type neuropeptides in *Aplysia* but there are also some similarities (e.g., inhibitory effect on feeding).

In conclusion, the discovery of similarities and differences in the functions of the GnRH-type and CRZ-type signaling systems in the starfish *A. rubens*, as reported here, is consistent with findings from other animals. However, clearly there is still much to be learnt about the physiological roles of the GnRH/CRZ-type neuropeptide signaling systems in starfish. The functional characterization of neuropeptides is dependent on the physiological and/or behavioral assays employed to investigate their roles. Here, we employed bioassays for muscle contractility, revealing that ArGnRH and ArCRZ are both myostimulatory peptides. This finding, combined with anatomical analysis of their expression patterns, represents a significant first advance in characterizing GnRH/CRZ-type neuropeptide signaling in echinoderms. In the future use of other bioassays may reveal additional functions of GnRH/CRZ-type neuropeptides in *A. rubens*. Furthermore, the findings reported here provide a basis for analysis of GnRH/CRZ-type neuropeptide signaling in other starfish species; for example, in the starfish species *P. pectinifera* and *Acanthaster planci* (the crown-of-thorns starfish), which are established or emerging model systems for neuropeptide research (69, 85–87). Looking beyond the class Asteroidea, orthologs of ArGnRH and ArCRZ have been identified in other echinoderms, including the sea urchin *Strongylocentrotus purpuratus* (88) and the brittle star *Ophionotus victoriae* (89) and therefore it will be of interest to investigate the expression and actions of GnRH/CRZ-type neuropeptides in these and/or other echinoderm species. The five extant echinoderm classes, the Asteroidea, Ophiuroidea, Echinoidea, Holothuroidea, and Crinoidea diverged during the Cambrian and Ordovician eras in a period estimated to be between 475 and 509 million years ago (90). Over the long period of evolutionary time that has elapsed since, there may have been clade-specific specialization in GnRH/CRZ-type neuropeptide function and therefore, a broad physiological/behavioral perspective may be necessary in selecting assays that reveal the actions of GnRH/CRZ-type neuropeptides in echinoderms.

## Ethics Statement

Approval by an ethics committee was not required for this work because experimental work on starfish is not subject to regulation.

## Author Contributions

This study was conceived by MRE and ST. ST obtained experimental data. ME generated the trichrome-stained sections of *A. rubens* and developed experimental methods for the anatomical studies. ST and MRE analyzed and interpreted data and wrote the article.

## Conflict of Interest Statement

The authors declare that the research was conducted in the absence of any commercial or financial relationships that could be construed as a potential conflict of interest.
