# Supplementary material for: Functional Characterization of Paralogous Gonadotropin-Releasing Hormone-Type and Corazonin-Type Neuropeptides in an Echinoderm
Source: Front Endocrinol (Lausanne). 2017 Sep 29;8:259. doi: 10.3389/fendo.2017.00259 (PMC5626854; doi:10.3389/fendo.2017.00259)
Supplement: Supplementary file 1 [file Data_Sheet_1.PDF]

*Supplementary Material*

# Functional Characterization of Paralogous GnRH-Type and Corazonin-Type Neuropeptides in an Echinoderm

**Shi Tian, Michaela Egertová, Maurice R. Elphick** *

*** Correspondence:** Maurice R. Elphick: m.r.elphick@qmul.ac.uk

# 1 Supplementary Figures and Tables

## 1.1 Supplementary Figures

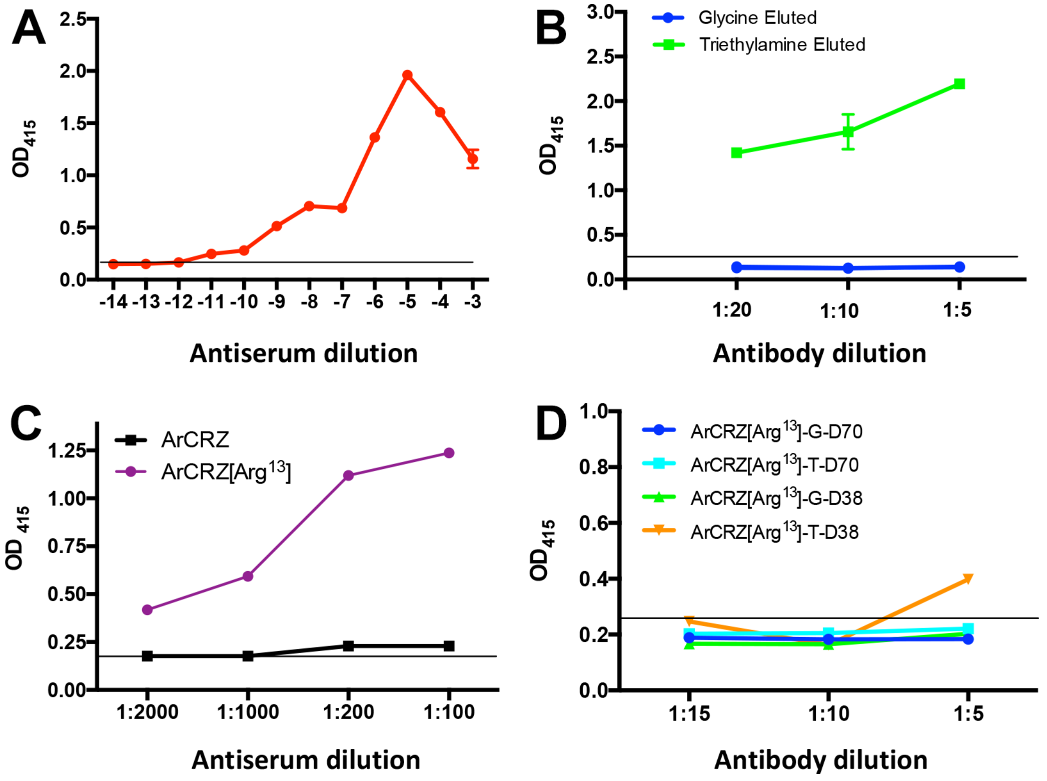


**Supplementary Figure 1.** Characterisation of antisera and affinity-purified antibodies using an enzyme-linked immunosorbent assay (ELISA). **(A)**. Incubation of an antiserum to ArGnRH (red) at dilutions between $10^{-3}$ and $10^{-14}$ with 0.1 nmol of antigen peptide per well reveals that the antigen is detected at well above the background (black) optical density with dilutions from $10^{-3}$ to $10^{-9}$. **(B)**. Incubation of affinity-purified antibodies with 0.1 nmol of antigen peptide per well reveals that antibodies to the antigen peptide can be detected in the triethylamine eluate but not in the glycine eluate. **(C)**. Incubation of an antiserum to ArCRZ (blue) at dilutions between 1:100 and 1:2000 with 0.1 nmol of antigen peptide per well does not reveal the presence of antibodies to the antigen peptide. However, incubation of an antiserum to ArCRZ[$Arg^{13}$] (purple) at dilutions between 1:100 and 1:2000 with 0.1 nmol of antigen peptide per well reveals the presence of antibodies to the antigen peptide. **(D)**. Incubation of affinity-purified antibodies to ArCRZ[$Arg^{13}$] with 0.1 nmol of antigen peptide per well reveals that antibodies to the antigen peptide cannot be detected in the triethylamine eluate or the glycine eluate of the day 38 or the day 70 antiserum bleeds, with the exception of the triethylamine eluate of the day 38 antiserum where a signal just above background levels is detected

but only with the 1:5 dilution. In all graphs, the data points represent mean values (± SEM) from at least three replicates.
